# Ferroptosis and metabolic reprogramming in the immunosuppressive microenvironment of glioblastoma: emerging mechanisms and novel strategies

**DOI:** 10.3389/fimmu.2026.1775148

**Published:** 2026-04-14

**Authors:** Liang Han, Zhengming Wang, Yichuan Jiang, Haoyu Zhou, Haixia Zhou

**Affiliations:** 1 Department of Pathology, China-Japan Union Hospital of Jilin University, Changchun, Jilin, China; 2 Trauma Center, China-Japan Union Hospital of Jilin University, Changchun, Jili, China; 3 Department of Pharmacy, China-Japan Union Hospital of Jilin University, Changchun, Jilin, China; 4 China-Japan Union Hospital of Jilin University, Changchun, Jilin, China; 5 Department of VIP Unit, China-Japan Union Hospital of Jilin University, Changchun, Jilin, China

**Keywords:** ferroptosis, glioblastoma, immunosuppressive microenvironment, immunotherapy, metabolic reprogramming

## Abstract

Glioblastoma (GBM) is the most common and aggressive primary brain tumor in adults. GBM often exhibits resistance to conventional apoptosis-inducing therapies, and its immunosuppressive microenvironment limits the efficacy of existing treatments. Ferroptosis is an iron-dependent, lipid peroxidation-driven form of cell death. The unique metabolic reprogramming in GBM, including dysregulated iron metabolism, abnormal lipid metabolism, and imbalanced antioxidant defenses, collectively determines the susceptibility of tumor cells to ferroptosis. There is a bidirectional regulatory relationship between ferroptosis and the tumor immune microenvironment (TIME). Ferroptosis can release damage-associated molecular patterns and activate dendritic cells, thereby enhancing antitumor immunity. Simultaneously, the functional state of immune cells directly influences the progression of ferroptosis. Targeting ferroptosis can enhance the efficacy of temozolomide (TMZ) and increase radiosensitivity. Nanodelivery systems can overcome blood–brain barrier limitations, enabling the co-delivery of ferroptosis inducers and immunomodulators. The combination of ferroptosis with immune checkpoint blockade can reverse the suppressive TIME. This review systematically summarizes the mechanisms by which ferroptosis regulates the suppressive TIME of GBM; the application of ferroptosis-targeting strategies (including ferroptosis inducers, immunotherapy, and targeted nanomaterials) in GBM treatment; and prospects for clinical translation. Targeting ferroptosis provides a new direction for modulating the suppressive TIME of GBM and developing novel therapeutic strategies for GBM.

## 1 Introduction

Glioblastoma (GBM) is the most common and aggressive primary malignant brain tumor in adults, with an incidence of approximately 3–5 cases per 100, 000 people, accounting for approximately 49% of all malignant brain tumors, and is associated with extremely high recurrence and mortality rates (1, 2). In regions such as China, the burden of central nervous system (CNS) tumors is increasing, reflecting the impact of improved diagnosis and an aging population (3). Despite the current standard treatments combining maximal safe resection, radiotherapy, and temozolomide (TMZ) chemotherapy, the prognosis for GBM patients remains extremely poor, with a median survival of approximately 12–15 months and an average 5-year survival rate of 13.8% (4–6). Owing to the overexpression of O6-methylguanine methyltransferase (MGMT) and/or the activation of DNA repair pathways in GBM cells, approximately 50% of GBM patients do not respond to TMZ therapy (7). Additionally, the highly heterogeneous microenvironment, rapid proliferation capacity, and high invasiveness of GBM can lead to apoptosis resistance in tumor cells, ultimately resulting in treatment failure or recurrence (8).

Compared with normal brain tissue, GBM cells exhibit significant metabolic alterations (e.g., the Warburg effect) and changes in key metabolic pathways, including fatty acid synthesis, glutamine metabolism, and the tricarboxylic acid (TCA) cycle (9). Furthermore, chemotherapy and radiotherapy induce DNA damage directly or indirectly through the generation of reactive oxygen species (ROS). However, GBM cells have a high metabolic rate and produce elevated levels of ROS, and their metabolic adaptations play a crucial role in resistance to oxidative stress-induced cell death. GBM cells utilize ROS to regulate multiple signaling pathways that control cellular stability, influence the cell cycle, and ultimately drive tumor progression and drug resistance (10). Therefore, metabolic reprogramming is recognized as a key mechanism in GBM development, progression, and treatment resistance.

Ferroptosis is a cell death mechanism that is dependent on the accumulation of iron ions and lipid peroxidation and is distinct from apoptosis, necrosis, and autophagy. Its core molecular mechanisms involve the collapse of the antioxidant defense system, particularly the inactivation of glutathione peroxidase 4 (GPX4), inhibition of the cystine/glutamate antiporter (System Xc^-^), and dysregulation of the ferroptosis suppressor protein 1 (FSP1) pathway (11). GPX4 relies on glutathione (GSH) to neutralize lipid peroxides. System Xc^-^ supports GPX4 function by maintaining the supply of cysteine and GSH, whereas FSP1 independently inhibits lipid peroxidation by reducing the level of ubiquinone (CoQ10), thereby preventing ferroptosis (12). When GPX4 is inactivated or when GSH is depleted in GBM cells, lipid peroxides cannot be effectively cleared, leading to membrane structural damage and triggering ferroptosis (13, 14). For example, the ferroptosis inducer RSL3 did not significantly affect the viability of T98G cells in the control group. Knockdown of selenoprotein P (SeP) downregulates GPX4 expression, enabling RSL3 to significantly inhibit T98 cell viability (15). The knockdown of heat shock protein 27 (HSP27) can upregulate the expression of ACSL4, one of the key enzymes in the lipid peroxidation pathway, increasing the levels of MDA and Fe^2+^, thereby promoting ferroptosis and suppressing tumor growth (16). These findings suggest that modulating key ferroptosis-related signaling molecules can increase susceptibility to ferroptosis, representing a potentially effective strategy for antitumor therapy in GBM.

**Figure 1:**
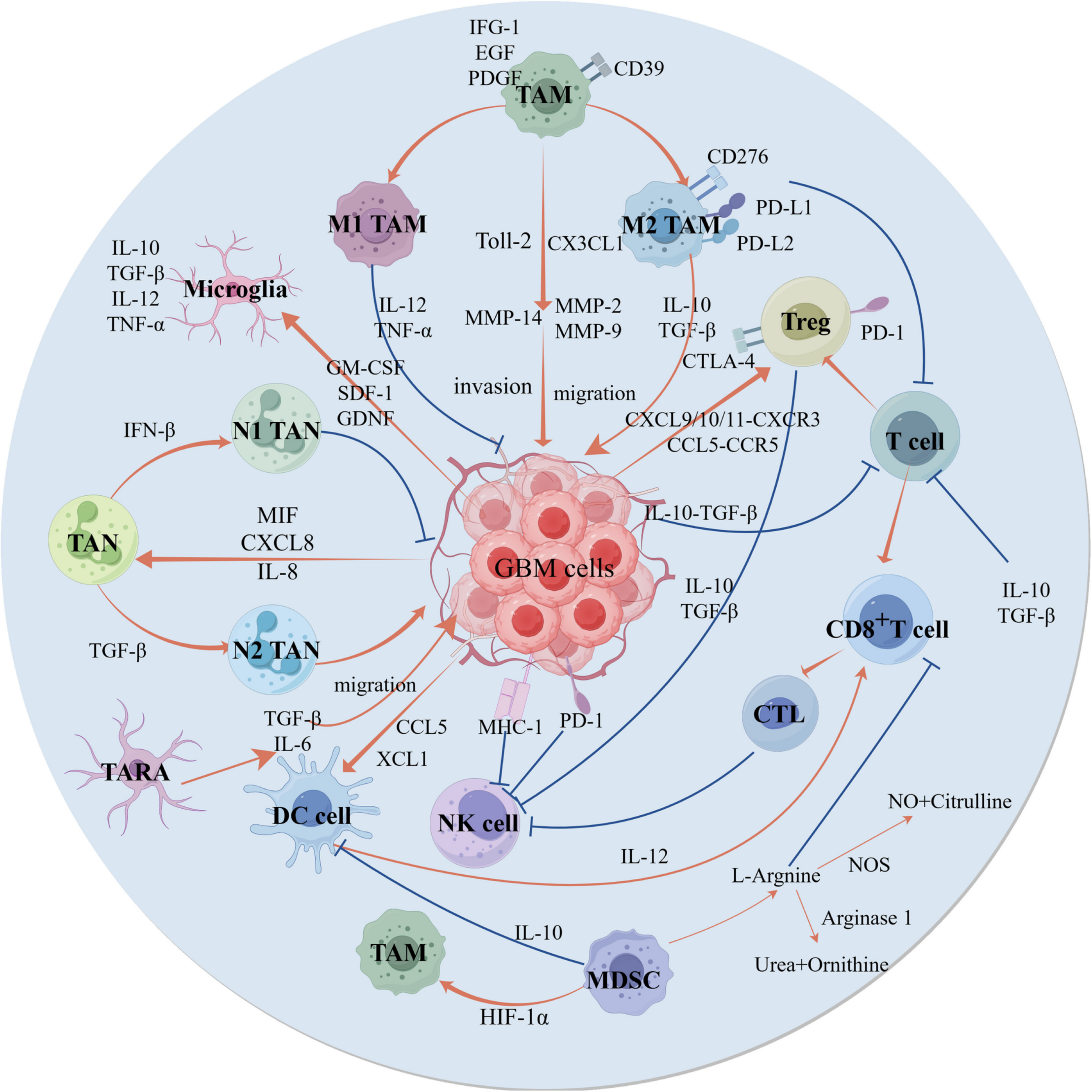
Cellular alterations and interactions in the GBM immune microenvironment. This image systematically illustrates the changes and interactions of immune cells in the TIME of GBM. The GBM tumor microenvironment (TME) primarily comprises tumor-promoting M2-type TAMs, N2-type TANs, Tregs, and tumor-suppressive M1-type TAMs, N1-type TANs, CD8+ T cells, and CTLs. TAMs express Toll-2 and CX3CX1, which promote the release of MMP-14 (promoting GBM invasion), MMP-2, and MMP-9 (promoting GBM migration). Among them, M1-type TAMs release IL-12 and TNF-α to exert tumor-suppressive effects, while M2-type TAMs release IL-10 and TGF-β to exert tumor-promoting effects. T cells differentiate into Tregs and CD8+ T cells. Tregs are recruited to the GBM TIME via the cytokines CXCL9/10/11-CXCR3 and CCL5-CCR5. CD8+ T cells differentiate into CTLs and inhibit NK cells, while IL-10 and TGF-β released by GBM cells suppress T cell activity. In GBM, TANs differentiate into tumor-suppressive N1-type TANs via IFN-β and tumor-promoting N2-type TANs via TGF-β. TANs are attracted to the GBM TIME through MIF, CXCL8, and IL-8. MDSCs inhibit CD8+ T cell activity by increasing the catabolism of L-arginine and suppress DC cell maturation by releasing IL-10. Astrocytes promote GBM migration by secreting IL-6 and TGF-β. These interactions among immune cells constitute the GBM TME. CTL, Cytotoxic T Lymphocyte; CX3CL1, C-X3-C motif chemokine ligand 1; DC, Dendritic Cell; GBM, Glioblastoma; GDNF, Glialcellline-derived neurotrophic factor; GM-CSF, Granulocyte-Macrophage Colony Stimulating Factor; IL-6, Interleukin 6; IL-10, Interleukin 10; MDSC, Myeloid-derived suppressor cells; MHC-1, Major histocompatibility complex 1; MMP-2, Matrix Metallopeptidase 2; MMP-9, Matrix Metallopeptidase 9; MMP-14, Matrix Metallopeptidase 14; NK, Natural Killer cells; NOS, Nitricoxide synthase; PD-1, Programmed death 1; TAM, Tumor-associated macrophage; TAN, Tumor associated neutrophils; TARA, Tumor-associated reactive astrocytes; TGF-β, Transforming growth factor-β; TME, Tumor immune microenvironment; TNF-α, Tumor-Necrosis Factor-alpha; Toll-2, Toll-like receptors 2; Treg, Tregulatory T cell; SDF-1, Stromal Cell-derived Factor 1. (Created by Figdraw).

The tumor immune microenvironment (TIME) of GBM is characterized by highly immunosuppressive characteristics, marked by the polarization of tumor-associated macrophages (TAMs) toward the M2 phenotype, T-cell functional exhaustion, excessive infiltration of regulatory T cells (Tregs), and high expression of the immune checkpoint molecules PD1/PD-L1 (17, 18). (**Figure 1**) The unique TIME of GBM not only promotes tumor cell growth and invasion but also limits the efficacy of existing chemotherapy and radiotherapy (19, 20). TAMs are the most abundant immune cell population in GBM, where “M2” polarized TAMs mediate tumor progression and therapeutic resistance through the secretion of immunosuppressive cytokines such as IL-10 and TGF-β. Single-cell and multiomics analyses by Kloosterman et al. revealed a subset of TAMs characterized by metabolic reprogramming, termed lipid-laden macrophages (LLMs). LLMs exhibit significant cholesterol accumulation and immunosuppressive properties. These LLMs transfer myelin-derived lipids to cancer cells in an LXR/Abca1-dependent manner, thereby fuelling the increased metabolic demands of mesenchymal GBM (21). CD8^+^ T-cell exhaustion and Treg enrichment further constrain cytotoxic T lymphocyte (CTL) activity, impairing antitumor immunity and facilitating immune escape (22). GBM cells engage in extensive interactions with immune cells, modulating cellular metabolism and function through the release of extracellular vesicles, cytokines, chemokines, and other bioactive mediators, thereby altering the TIME (23, 24). For instance, macrophages mediate the transition of GBM cells to a mesenchymal-like (MES-like) state via the release of oncostatin M (OSM). Conversely, MES-like GBM cells are associated with enhanced mesenchymal programs in macrophages and increased numbers of cytotoxic T cells. This study highlights the broad remodeling of the TIME of GBM, with potential therapeutic implications (25).

Metabolic alterations are considered key drivers in regulating the suppressive TIME of GBM. For example, the accumulation of lactate and lipid droplets in the microenvironment may modulate immune cell function, thereby influencing GBM progression (26, 27). Interestingly, ferroptosis is extensively involved in the complex regulatory network between GBM cells and immune cells. Ferroptosis in tumor cells can release damage-associated molecular patterns (DAMPs), thereby inducing immunogenic cell death (ICD), activating DC and CTL responses, and initiating adaptive immune responses (28–30). On the other hand, ferroptosis in immune cells can weaken antitumor immunity (31, 32). Ferroptosis-related genes (FRGs) signatures are closely associated with the immune infiltration status, prognostic risk, and drug sensitivity in patients with GBM (33, 34). These studies suggest that ferroptosis may provide new therapeutic avenues for GBM through the modulation of tumor metabolism and TIME. Therefore, this article aims to systematically discuss how metabolic alterations regulate the ferroptosis process and to highlight the interactions between ferroptosis and the immunosuppressive microenvironment. By integrating the current research, we further analyzed potential therapeutic strategies targeting ferroptosis and the TIME. This exploration not only deepens the understanding of the biological behavior of GBM but also provides a theoretical foundation for the development of novel combination therapies.

## 2 Ferroptosis susceptibility and resistance in GBM

### 2.1 Disorders of iron homeostasis

**Figure 2:**
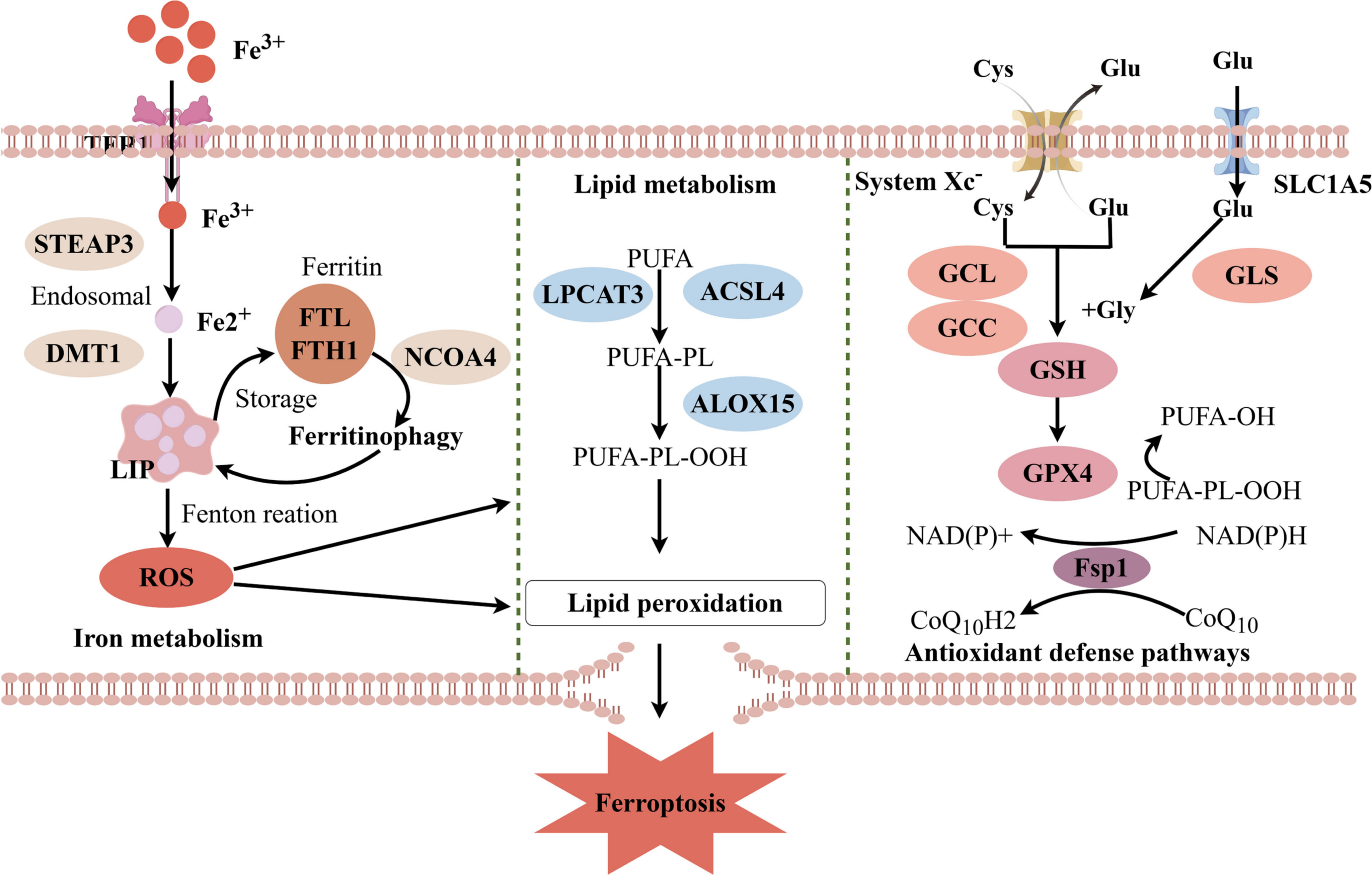
Regulatory network of metabolic reprogramming and ferroptosis in GBM. This image systematically illustrates the core molecular network through which metabolic reprogramming regulates ferroptosis in glioblastoma. The network primarily comprises three modules: dysregulated iron metabolism (left), lipid metabolic remodeling (center), and an imbalanced antioxidant defense system (right). In the iron metabolism module, increased iron uptake mediated by TFR1/DMT1, aberrant storage via ferritin (FTH/FTL), and NCOA4-driven ferritinophagy collectively lead to the expansion of the labile iron pool (LIP). This provides the substrate for the Fenton reaction. In the lipid metabolism module, the ACSL4/LPCAT3 pathway promotes the esterification of polyunsaturated fatty acids (PUFAs) into membrane phospholipids, laying the foundation for lipid peroxidation. In the antioxidant module, impaired System Xc^-^ (SLC7A11/SLC3A2) function or GPX4 inactivation results in glutathione (GSH) depletion and the failure to clear lipid peroxides. Concurrently, the FSP1/CoQ10 axis provides GPX4-independent compensatory protection. These pathways act synergistically, ultimately driving the execution of ferroptosis through irrepressible lipid peroxidation. ACSL4, Acyl-CoA Synthetase Long Chain Family Member 4; CoQ10, Coenzyme Q10; DMT1, Divalent Metal Transporter 1; FSP1, Ferroptosis Suppressor Protein 1; FTH, Ferritin Heavy Chain; FTL, Ferritin Light Chain; GPX4, Glutathione Peroxidase 4; GSH, Glutathione; GSSG, Glutathione Disulfide; LIP, Labile Iron Pool; LPCAT3, Lysophosphatidylcholine Acyltransferase 3; NCOA4, Nuclear Receptor Coactivator 4; PUFA, Polyunsaturated Fatty Acids; ROS, Reactive Oxygen Species; SLC3A2, Solute Carrier Family 3 Member 2; SLC7A11, Solute Carrier Family 7 Member 11; STEAP3, Six-Transmembrane Epithelial Antigen of the Prostate 3; TFR1, Transferrin Receptor 1. (Created by Figdraw).

Cancer cells undergo significant metabolic changes in hostile environments characterized by nutrient deprivation, poor vascularization, and immune infiltration. The versatile electron transfer capability of iron makes it a multifunctional cofactor involved in countless biochemical reactions critical for cellular homeostasis, including cellular respiration and DNA replication (35). Cancer cells employ various mechanisms to increase iron bioavailability, thereby promoting tumor growth, altering cell morphology and metastasis, and maintaining tumor stem cells and the TIME (36). Although iron itself can readily participate in redox reactions, enabling essential processes, its reactivity also generates ROS. Consequently, cancer cells further rely on antioxidant mechanisms to withstand this stress (35). These alterations increase the susceptibility of tumor cells to ferroptosis under certain conditions. For instance, compared with cells with epithelial traits, cancer cells with mesenchymal characteristics are generally more prone to ferroptosis, and drug-resistant cancer cells undergoing epithelial–mesenchymal transition (EMT) become more vulnerable to ferroptosis inducers (37). In GBM, this regulatory mechanism is disrupted, leading to a significant expansion of the intracellular labile iron pool, which provides ample substrate for the Fenton reaction, thereby driving lipid peroxidation and ferroptosis (38). Tong et al. reported that in U87 and U251 cells with increased iron uptake, the overexpression of transferrin receptor 2 (TFR2), a protein responsible for cellular iron uptake, is associated with increased susceptibility to ferroptosis (39). Dysregulated iron metabolism not only affects tumor cell survival and proliferation but also shapes the unique biological behavior of tumors by altering redox balance (**Figure 2**).

In GBM cells, the overactivation of iron uptake mechanisms is a key factor leading to iron overload. Studies have shown that the expression of transferrin receptor protein 1 (TFRC) and divalent metal transporter 1 (DMT1) is significantly upregulated in GBM (40), promoting the continuous influx of extracellular iron. This enhanced iron uptake aligns with the high proliferation demands of tumor cells but also introduces the risk of iron toxicity. Wu et al. reported that the expression of TFRC is significantly correlated with that of ferroptosis-related genes (such as HSPA1, SLC7A11, and NFE2L2) and that there are notable associations between TFRC and immune cells (CD8+ T cells and TAMs), suggesting that TFRC is a potential therapeutic target (41). Additionally, Santiago et al. constructed conditional gene knockout mice to study iron metabolism in Schwann cells. DMT1 gene knockout disrupted cell formation and myelination, resulting in iron deficiency, whereas the absence of DMT1 also affected transferrin receptor protein 1 (TfR1) expression (42). Preclinical research by Chitambar et al. revealed that interfering with iron metabolism in GBM cell lines (U-87 MG and D54) and multiple GBM stem cell (GSC) lines using gallium maltolate (GaM) resulted in upregulated TfR1 expression after GaM treatment. These findings confirmed that restricting tumor iron uptake significantly inhibits mitochondrial function and nucleotide synthesis, thereby suppressing GBM tumor growth (43).

Dysregulation of the iron storage system also plays a significant role in iron metabolism dysregulations in GBM. The ferritin complex, composed of a light chain (FTL) and a heavy chain (FTH1), is responsible for safely storing intracellular iron and preventing free iron-induced oxidative damage. However, the expression of FTH1 and FTL in GBM is often abnormal, and their functions can be altered because of disrupted transcriptional regulation or reprogramming of iron metabolism pathways (44, 45). Huan et al. reported that in TP53-mutant GBM, dysregulation of FTH leads to disruption of iron homeostasis, thereby increasing cellular susceptibility to ferroptosis (46). Additionally, iron-responsive element-binding protein 2 (IREB2), a member of the iron regulatory protein family, regulates the expression of iron-related genes such as TfR1, FTH1, and FTL. Its dysregulation may result in imbalances in iron uptake and storage (47). Impaired ferritin function not only directly increases the size of the labile iron pool but also increases the vulnerability of cells to oxidative stress by affecting the dynamic equilibrium of iron (48).

Ferritinophagy, as a selective autophagy process, plays a key role in iron metabolism disorders in GBM. Nuclear receptor coactivator 4 (NCOA4) is a critical regulator of ferritinophagy, which promotes the degradation of ferritin and the release of iron ions by mediating the binding of ferritin to autophagosomes (49). Studies have shown that TRIM7 affects ferritinophagy and ferroptosis processes by regulating the stability of NCOA4 through ubiquitination, revealing a potential therapeutic target (50). Similarly, Kuno et al. reported that NCOA4 can form condensates under iron overload conditions, regulating the rate of ferritin degradation to modulate intracellular iron homeostasis (49). This increase in ferritinophagy is closely associated with the malignant progression of GBM. Therefore, the link between iron metabolism disorders and ferroptosis susceptibility provides a new perspective for GBM treatment.

### 2.2 Lipid metabolism remodeling drives ferroptosis

The malignant progression of GBM is accompanied by significant lipid metabolic remodeling, a process that creates the necessary molecular conditions for the occurrence of ferroptosis. The core feature of lipid metabolic reprogramming is the abnormal activation of the PUFA metabolic pathway, particularly through the synergistic action of long-chain acyl-CoA synthetase 4 (ACSL4) and lysophosphatidylcholine acyltransferase 3 (LPCAT3), which promote the esterification of PUFAs into membrane phospholipids (11). ACSL4 is responsible for catalyzing the activation of PUFAs, forming acyl-CoA derivatives, whereas LPCAT3 further integrates these activated fatty acids into membrane phospholipids, especially phosphatidylethanolamine (51, 52). This cascade reaction leads to a significant increase in the content of PUFAs such as arachidonic acid and adrenic acid in the cell membrane, providing abundant substrates for subsequent lipid peroxidation reactions.

ACSL4 is a lipid metabolism-related enzyme that catalyzes the activation of PUFAs, thereby participating in the process of lipid peroxidation. In GBM, the expression level of ACSL4 is closely related to the sensitivity of tumor cells to ferroptosis and is also positively correlated with the sensitivity of GBM cells to ferroptosis inducers. Studies have shown that the expression of ACSL4 significantly increases during GBM recurrence and that its upregulation markedly enhances the sensitivity of tumor cells to oxidative stress (53). Additionally, preclinical experiments by Bao et al. revealed that the negative regulation of ACSL4 by miR-670-3p can inhibit ferroptosis in GBM cells and affect their proliferative phenotype. This mechanism could serve as a potential therapeutic strategy for GBM (54). Medium-chain acyl-CoA dehydrogenase (MCAD) is an enzyme involved in the oxidation of medium-chain fatty acids, catalyzing their β-oxidation to generate energy and eliminate toxic metabolites. In GBM, its expression is significantly upregulated, indicating that tumor cells are highly dependent on fatty acid metabolism (55). RNAi screening experiments revealed that MCAD deficiency leads to the accumulation of medium-chain fatty acids, causing harmful metabolic effects, and in GBM models, it also results in mitochondrial damage and cell death (55).

The execution of lipid peroxidation primarily relies on the catalytic activity of the lipoxygenase (ALOX) family, with ALOX15 playing a critical role in GBM. ALOXs can directly oxidize PUFA phospholipids on cell membranes, initiating a chain reaction of lipid peroxidation, ultimately leading to the disruption of cell membrane integrity (56). Studies have indicated that ALOX15 is upregulated in GBM cells and that its activation is closely associated with mitochondrial dysfunction. This upregulation increases the ratio of oxidative to nonoxidative lipids, triggering ferroptosis through the accumulation of lipid ROS. Additionally, small activating RNA (saRNA)-mediated expression of ALOX15 can significantly promote ferroptosis in GBM cells and inhibit tumor growth (57). Furthermore, the metabolic product of ALOX15, 13-HODE, exhibits protumor effects in GBM cells, further emphasizing the importance of lipid metabolism in GBM progression (58). In addition to ALOXs, other lipid metabolism enzymes, such as fatty acid desaturase 2 (FADS2) and lipoprotein-associated phospholipase A2 (PLA2G7), are involved in regulating PUFA biosynthesis. FADS2 modulates the synthesis of PUFAs in membrane phospholipids, thereby influencing cellular sensitivity to ferroptosis, and inhibition of FADS2 may enhance the therapeutic efficacy of ferroptosis induction (59). PLA2G7, a key gene of Lp-PLA2, indirectly participates in the regulation of ferroptosis by modulating intracellular phospholipid metabolism (60). The combined action of these enzymes leads to the accumulation of lipid peroxidation products within cells. When this accumulation surpasses the cell’s intrinsic antioxidant capacity, the ferroptosis program is triggered.

Notably, the genetic background of GBM may also influence ferroptosis susceptibility through lipid metabolism remodeling. Cyclin-dependent kinase inhibitor 2A (CDKN2A) is a crucial tumor suppressor gene encoding the p14/p16 protein and is involved in cell cycle regulation. On the basis of multimodel and multiomics studies, Minami et al. revealed that CDKN2A deletion reshaped the lipidome of GBM, redistributing oxidative PUFAs to distinct lipid compartments, thereby increasing lipid peroxidation levels and significantly increasing sensitivity to ferroptosis (61). CDKC2A deletion, particularly the loss of p14/16 expression, increases lipid peroxidation and ferroptosis susceptibility in GBM. p16 deletion leads to more pronounced ferroptosis in GBM, whereas GPX4 inhibition reduces the tumor burden of CDKN2A-deficient GBM *in vivo* (61). This discovery suggests a functional interplay between lipid metabolic remodeling and genetic mutations, the outcome of which may determine GBM’s functional choices in lipid metabolism. TRAF3 (TNF receptor-associated Factor 3), a member of the TNF receptor-associated factor family, participates in regulating immune responses and apoptosis. Preclinical research by Zeng et al. indicated that the expression of TRAF3 is frequently suppressed in GBM because of promoter hypermethylation. It interacts with enoyl-CoA hydratase 1 (ECH1) and mediates K63-linked ubiquitination of ECH1 at Lys214, hindering its mitochondrial translocation. Overexpression of this gene enhances PUFA metabolism and fatty acid oxidation (FAO), reduces lipid peroxidation, and protects GBM cells from ferroptosis (62). TRAF3 overexpression conversely promoted ECH1 ubiquitination, inhibited FAO, increased lipid peroxidation, and induced ferroptosis. ECH1 knockout causes mitochondrial damage and suppresses tumor formation, confirming the critical role of the TRAF3/ECH1 axis in ferroptosis (62). Therefore, lipid availability in the TIME is considered a key exogenous regulator of ferroptosis sensitivity.

### 2.3 Imbalance of the antioxidant defense system

The occurrence of ferroptosis in GBM cells not only depends on the activation of death signaling but is also closely related to the functional imbalance of the endogenous antioxidant defense system. System Xc-, as a key cystine uptake channel, is composed of two subunits, SLC7A11 (also known as xCT) and SLC3A2. It mediates the uptake of extracellular cystine and the efflux of intracellular glutamate, providing a critical precursor for GSH biosynthesis (63). In GBM, the expression of System Xc- is typically upregulated, which helps tumor cells maintain high levels of GSH in harsh microenvironments, thereby enhancing their survival capacity (64). When system Xc- function is impaired, cystine uptake decreases, leading to restricted GSH synthesis, increased ROS accumulation, and weakened cellular antioxidant defense capacity, thereby inducing tumor cell death (65). This metabolic disorder creates favorable conditions for ferroptosis, as GSH depletion directly affects the activity of key downstream antioxidant enzymes. High expression of SLC7A11 in GBM cells significantly enhances their resistance to ferroptosis, and its activity is regulated by multiple signaling pathways. For example, LINC01088 can increase SLC7A11 expression through the HLTF/USP7 axis, thereby inhibiting ferroptosis in GBM (66). Additionally, targeting SIRT3 to indirectly suppress SLC7A11 expression by promoting mitophagy enhances GBM sensitivity to ferroptosis (67). Notably, Merlin/NF2 is a critical regulator of SLC7A11 expression in GBM cells. Under high cell density, it reduces SLC7A11 protein levels and inhibits cystine uptake by promoting SLC7A11 lysosomal degradation, enabling cell survival under glucose deprivation (68). Conversely, when the Merlin/NF2 gene is deleted, SLC7A11 protein levels and cystine uptake significantly increase, leading to increased cell death under glucose-deficient conditions and revealing the dynamic interplay between metabolic stress and antioxidant defense. These regulatory mechanisms collectively maintain the appropriate activity of System Xc-, ensuring the continuous synthesis of GSH.

As it is an essential cofactor of GPX4, the reduction in GSH directly weakens the ability of GPX4 to reduce the levels of lipid peroxides. The overexpression and knockdown of GPX4 can modulate the lethality of various ferroptosis inducers, suggesting that GPX4 is a key regulatory factor in ferroptosis-induced cancer cell death (69). Studies have shown that the overexpression of GPX4 significantly inhibits the growth of GBM cells, leading to cell cycle arrest in the G1/G0 phase and increasing the cell surface area and Young’s modulus, triggering abnormal subdiffusion phenomena (70). The Meng team highlighted through preclinical research that the expression of GPX4 is regulated by METTL3-mediated m6A methylation, which is in turn controlled by the C5aR1-ERK1/2 signaling axis. The inhibition of C5aR1 or METTL3 expression results in GPX4 downregulation and ferroptosis induction, underscoring its potential as a therapeutic target for GBM (71). Additionally, targeting the ERK1/2 pathway or METTL3 activity may destabilize GPX4 and induce ferroptosis in GBM cells. Furthermore, sensitivity to GPX4-dependent ferroptosis is closely linked to the heterogeneity of GBM cell states. Activation of the Notch pathway drives cells into a quiescent astrocyte-like state, characterized by elevated mitochondrial lipid peroxidation and increased ROS and GSH depletion, increasing their susceptibility to GPX4 inhibition and ferroptosis induction (72). GPX4 inhibitors can selectively eliminate these quiescent astrocyte-like GBM cells, which are often resistant to standard therapies, while p53 deficiency promotes a proliferative progenitor-like state in GBM cells (72). Depletion of GSH compromises cellular antioxidant capacity, leading to ROS accumulation and oxidative stress. Notably, in certain contexts, cysteine and homocysteine can directly support the antioxidant function of GPX4 independent of GSH synthesis, and this alternative pathway may provide temporary protection during GSH depletion (73).

However, when GSH levels decrease or GPX4 activity is inhibited, GBM cells can activate alternative antioxidant pathways to maintain redox homeostasis, among which the ferroptosis suppressor protein 1 (FSP1)/coenzyme Q10 (CoQ10) system is the most representative compensatory mechanism (74). FSP1 reduces CoQ10 to generate CoQ10H2 (hydrogenated coenzyme Q10), which acts as a potent inhibitor of lipid peroxidation, directly capturing lipid peroxyl radicals and halting the chain reaction of lipid peroxidation. Additionally, FSP1 further influences cellular sensitivity to ferroptosis by regulating cellular homeostasis and metal ion response systems. This mechanism is particularly critical in GBM cells, as GBM typically exhibits enhanced dependence on GSH metabolism and is prone to developing drug resistance. Studies have shown that FSP1 expression is significantly upregulated in GBM and that DNA methylation-regulated lncRNAs (such as RYR3-DT) can modulate the FSP1/CoQ10 axis to affect ferroptosis sensitivity in GBM cells (75). Specifically, silencing these lncRNAs suppresses FSP1 expression, thereby increasing GBM cell sensitivity to ferroptosis. Furthermore, when overexpressed in GBM, other regulatory factors, such as indoleamine 2, 3-dioxygenase 1 (IDO1), significantly increase GSH levels. Research has indicated that IDO1 affects SLC7A11 mRNA stability by regulating m6A methylation, indirectly participating in the antioxidant defense regulatory network (76). Moreover, apolipoprotein C1 (APOC1) enhances GSH synthesis and GPX4 activity by modulating KEAP1/NRF2 expression and upregulating cystathionine β-synthase (CBS) expression, thereby reducing ferroptosis in GBM cells (77). These intricate regulatory mechanisms collectively form a multilayered defense system that enables GBM cells to resist ferroptosis.

## 3 Interaction between ferroptosis and the TIME of GBM

### 3.1 Direct regulation of immune cell activity and function by ferroptosis

**Figure 3:**
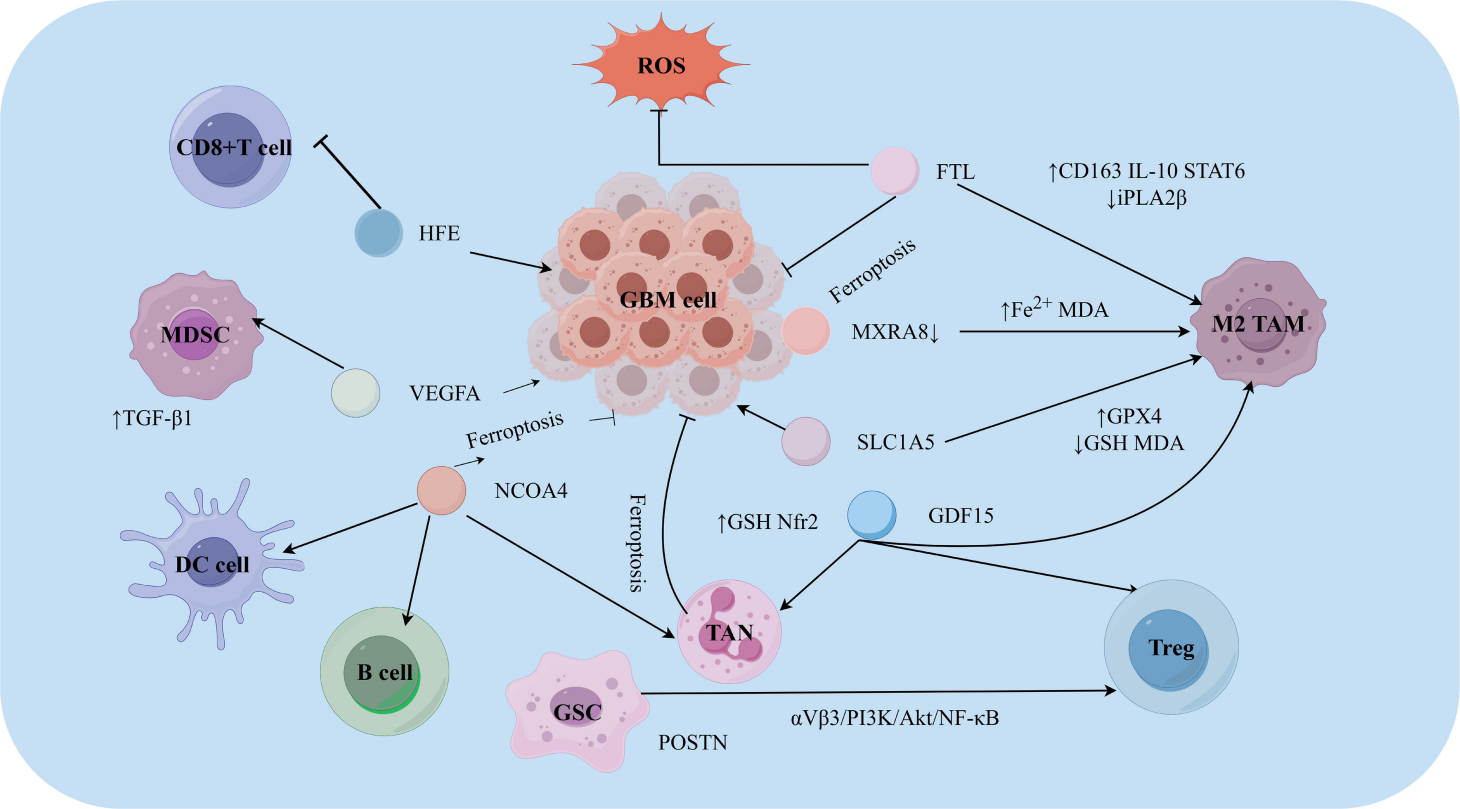
Functions and mechanisms of ferroptosis in the TIME of GBM. This image reveals the differential responses of various immune cells to ferroptosis within the glioblastoma microenvironment. The section illustrates immune cell populations exhibiting higher susceptibility to ferroptosis. These include M1 macrophages, myeloid-derived suppressor cells (MDSCs), dendritic cells (DCs), M2 macrophages, and CD8+ T cells. These cells maintain their functional states through distinct molecular mechanisms. They utilize unique protective mechanisms to survive under ferroptotic stress. Glioblastoma cells and the TIME interact with these immune cells through a complex signaling network. These interactions collectively shape the immunosuppressive TIME and influence the efficacy of anti-tumor immune responses. CD163, Cluster of Differentiation 163; GBM, Glioblastoma; GDF15, Growth Differentiation Factor 15; GPX4, Glutathione Peroxidase 4; GSH, Glutathione; IL-10, Interleukin-10; iPLA2β, Calcium-independent phospholipase A2β; MDA, Malondialdehyde; MDSC, Myeloid-Derived Suppressor Cells; MXRA8, Matrix Remodeling Associated 8; NCOA4, Nuclear Receptor Coactivator 4; NRF2, Nuclear factor erythroid 2-related factor 2; POSTN, Periostin; ROS, Reactive Oxygen Species; SLCA1A5, Solute Carrier Family A1 Member 5; STAT6, Signal Transducer and Activator of Transcription 6; TAM, Tumor-associated macrophage; TGF-β1, Transforming Growth Factor-beta 1; VEGFA, Vascular endothelial growth factor. (Created by Figdraw).

Recent studies have shown that ferroptosis plays a key regulatory role in the GBM TIME (**Figure 3**). Recent research has revealed that ferroptosis, through the release of lipid peroxidation products and DAMPs, can significantly affect the activity, phenotype, and function of tumor-infiltrating immune cells, thereby playing a dual role in tumor immune escape and immunotherapy response. For example, studies have shown that ferroptosis promotes T-cell infiltration in GBM, with the infiltration rate of CD4+ T cells increasing 20-fold and that of CD8^+^ T cells increasing 5-fold (78). Additionally, the status of isocitrate dehydrogenase (IDH) in GBM determines the central carbon metabolism process mediated by oncolytic herpes simplex virus (oHSV), influencing lipid peroxidation and ferroptosis and thereby affecting antitumor immunity (79). Programmed cell death (PCD) is intentionally induced cell death that alters the adjacent TIME during GBM progression. A bioinformatics analysis of 1, 750 patients revealed that compared with other histopathological subtypes, GBM consistently has higher PCD scores and that ferroptosis is enhanced in GBM (80). Furthermore, high ferroptosis scores correlate with the infiltration of immunosuppressive cells such as Tregs, neutrophils, and macrophages.

#### 3.1.1 CD8^+^ T cells

CD8^+^ T cells play a critical role in antitumor immunity, and ferroptosis indirectly affects the activity of CD8^+^ T cells by regulating the metabolic and immune evasion mechanisms of tumor cells. As the most enriched PCD process in GBM, ferroptosis induces immunosuppression. Studies have shown that the use of ferrostatin-1 (Fer-1, a ferroptosis inhibitor) can increase T-cell activity, and its combination with anti-PD1/L1 immunotherapy leads to the enrichment of CD8^+^ T cells in tissues, significantly increasing the immune infiltration of CD8^+^ T cells (80). Additionally, homeostatic iron regulators (HFEs) are crucial for GBM cell growth and survival, promoting the proliferation and survival of GBM tumor cells in a sex-specific manner and influencing CD8^+^ T-cell activity (81). These findings suggest that ferroptosis can affect the activity of CD8^+^ T cells and that HFEs can influence their activity in a sex-specific manner.

#### 3.1.2 Tregs

Treg cells are a subset of T cells with immunosuppressive functions that help tumors evade immune surveillance by inhibiting the activity of effector T cells, thereby promoting tumor growth. In GBM, the infiltration of Treg cells is closely associated with the immunosuppressive TME. Studies have shown that POSTN secreted by GSCs recruits microglia and upregulates CD70 expression through the αVβ3/PI3K/AKT/NF-κB pathway, thereby promoting the development and function of Treg cells and supporting the formation of an immunosuppressive TIME (82). Xu et al. reported that the loss of GPX4 leads to the accumulation of lipid peroxides in Treg cells, triggering ferroptosis, which weakens their immunosuppressive function and upregulates the production of IL-1β, promoting a TH17 response and thereby diminishing the inhibitory effect of Treg cells on tumor immunity (83). These findings suggest that selective modulation of the GPX4 axis may weaken the immunosuppressive microenvironment without damaging Treg cells.

#### 3.1.3 TAMs in GBM

TAMs are the most abundant nontumor cell population in the GBM microenvironment and are primarily classified into M1 and M2 phenotypes. M2-type TAMs possess protumor properties, weakening antitumor immune responses through the secretion of immunosuppressive factors such as IL-10 and TGF-β. Research by the Liu team revealed that M2-type macrophages are significantly enriched in GBM patients with high ferroptosis scores. In patients with high ferroptosis scores, the M1-type TAM marker CD86 is downregulated, while the M2-type TAM marker CD163 is upregulated. Further experiments in intracranial GBM xenograft mouse models demonstrated that ferroptosis leads to increased TAM infiltration and M2-type polarization. Treatment with the ferroptosis inhibitor Fer-1 significantly reduced the tumor M2/M1 ratio, indicating that ferroptosis inhibition promotes the polarization from M2 to M1 (80). Bioinformatics analysis revealed that FTL increases the expression of CD163, IL-10, and STAT6 (M2-type markers) and reduces iron overload and ROS generation, thereby inducing M2-type polarization and promoting ferroptosis (44). Further studies revealed that iPLA2β (favoring M1-type) is expressed at low levels in GBM and TAMs. FTL promotes ferroptosis by inhibiting iPLA2β expression in TAMs, thereby facilitating the polarization of TAMs toward the M2 phenotype (44). Matrix remodeling-associated protein 8 (MXRA8) was identified as a novel biomarker involved in ferroptosis and associated with glioma prognosis. MXRA8 is highly expressed in glioma tissues, and its knockdown inhibits the survival rate of glioma cells (e.g., T98G and U251 lines), leading to elevated intracellular iron ion and lipid peroxidation levels, accompanied by suppression of FTH1 and upregulation of NCOA4. MXRA8 knockdown significantly reduces M2-type TAM infiltration, and this effect is reversible by the ferroptosis inhibitor Fer-1 (84). Solute carrier family 1 member 5 (SLC1A5) is significantly overexpressed in GBM tissues and regulates ferroptosis sensitivity through a GPX4-dependent pathway. Its knockdown inhibits GBM cell proliferation and invasion and reduces ferroptosis sensitivity. Additionally, high SLC1A5 expression is correlated with immunosuppression, and its knockdown reduces TAM infiltration and M2 polarization (85). Notably, in macrophages and microglia, redox lipid reprogramming directly influences ferroptosis sensitivity. M1-type macrophages and microglia exhibit increased resistance to ferroptosis in proinflammatory environments, whereas the absence or inhibition of inducible nitric oxide synthase (iNOS) significantly increases their susceptibility to ferroptosis. Nitric oxide (NO•) donors can increase ferroptosis resistance in M2-type cells by modulating the nitro-oxidation of lipid peroxidation intermediates (86). The expression of growth differentiation Factor 15 (GDF15), a stress-responsive cytokine, is upregulated in GBM. Studies have shown that after radiotherapy, GDF15 promotes M2-type TAM infiltration, resulting in the formation of an immunosuppressive microenvironment (87). These studies demonstrate that ferroptosis affects the functional balance of TAMs in the immune microenvironment by altering their iron metabolism and lipid signaling pathways.

#### 3.1.4 MDSCs

Myeloid-derived suppressor cells (MDSCs) are a heterogeneous group of immature myeloid cells that are significantly expanded in GBM and promote tumor immune escape through the suppression of immune cells such as T cells (88). Studies have shown that in GBM, the overexpression of CD200 by GBM cells significantly promotes the recruitment of MDSCs (89). Additionally, the accumulation of MDSCs is closely associated with poor prognosis in GBM patients. Tian et al. reported that vascular endothelial growth Factor A (VEGFA) signaling promotes MDSC differentiation and accumulation, enhancing GBM immune escape. Moreover, MDSCs suppress the knockdown effect of VEGFA by secreting molecules such as TGF-β1, which has also been shown to regulate the sensitivity to ferroptosis (90). These findings suggest that in the TME, ferroptosis may weaken antitumor immunity by inducing functional activation of MDSCs.

#### 3.1.5 DC

Dendritic cells (DCs) are key antigen-presenting cells (APCs) that initiate primary T-cell responses. Reducing the release of GBM-derived exosomes (GDEs) can decrease lipid accumulation within DCs, lower their lipid peroxidation levels, and attenuate ferroptosis (91). Additionally, studies have shown that the overall abundance of DCs in GBM is increased and more significantly in IDH-mutant types and that dysfunctional DCs limit antigen-specific T-cell responses in GBM (92). A study investigating whether the addition of a dendritic cell vaccine loaded with autologous tumor lysate (DCVax-L) to standard-of-care (SOC) therapy prolongs the survival of GBM patients revealed that vaccinated patients exhibited improved survival rates and that CD8^+^ T cells could infiltrate into the GBM after DC vaccination (93). These findings suggest that GDEs may inhibit mature DC-mediated responses by inducing ferroptosis.

#### 3.1.6 NK cells

In GBM, the infiltration and functional status of NK cells are closely associated with tumor progression and treatment response. The abundance and activity of NK cells may be influenced by various factors, including FRG expression. Research has indicated that FRG-based prognostic models can predict the survival rate of GBM patients and are closely linked to NK cell infiltration in the TIME (94). Furthermore, GBM patients with a high cell death index (CDI) often exhibit reduced natural killer (NK) cell abundance, which may correlate with poor survival outcomes (95).

#### 3.1.7 Neutrophils

Pathologically activated neutrophils (PMNs), known as myeloid-derived suppressor cells (PMN-MDSCs), are major negative regulators of antitumor immunity. Studies have shown that PMN-MDSCs in the TIME undergo spontaneous death due to ferroptosis. In immunocompetent mice, inhibiting ferroptosis can eliminate the suppressive activity of PMN-MDSCs, slow tumor progression, and synergize with immune checkpoint blockade to suppress tumor growth (96). Additionally, research has shown that neutrophil-induced ferroptosis promotes tumor necrosis in GBM and induces tumor cell death *in vitro*. The overexpression of GPX4 or the depletion of ACSL4 in GBM cells inhibits tumor necrosis and invasion (97). LC3-associated phagocytosis is a novel noncanonical form of autophagy. Studies have revealed that LC3-associated neutrophil phagocytosis triggers ferroptosis in tumor cells within GBM. This process can be blocked by inhibiting the PI3K complex containing Vps34-UVRAG-RUBCN, and targeting this pathway may improve the prognosis of GBM (98).

Ferroptosis not only alters the physiological activity of immune cells but also plays a critical role in the immune therapy response. Clinical data indicate that the expression of ferroptosis-related genes (such as NFE2L2 and NOX4) is positively correlated with the infiltration levels of immunosuppressive cells, such as macrophages, Tregs, and Th2 cells and that high expression of these genes predicts immune escape and poor prognosis (99). In mouse models, Fer-1 treatment can increase the number of tumor Th1 cells and memory T cells, increase T-cell activity, and upregulate inhibitory immune checkpoints (PD1, CTLA4, TIM3, and LAG3) in T cells. Further analysis revealed that T-cell deficiency can counteract the tumor growth deceleration caused by the combination of Fer-1 and PD1/L1 blockade, suggesting that the effect of Fer-1 on tumor growth may depend on the presence of T cells (80). Additionally, inhibiting MS4A4A expression can convert M2-type TAMs to the M1 phenotype and reduce the infiltration of M2-type TAMs through the ferroptosis pathway, thereby enhancing the response of GBM to PD-1 immunotherapy (100).

In summary, ferroptosis regulates the immune microenvironment in GBM through multiple mechanisms: promoting DC activation and CD8+ T-cell responses, modulating the direction of TAM polarization, maintaining the immune suppression stability of Tregs, and potentially enhancing immune escape through MDSCs. This complex interaction network reflects that ferroptosis can act as both an immune activation signal and a potential inducer of immune tolerance.

### 3.2 Feedback and resistance of the immune microenvironment to ferroptosis

#### 3.2.1 Extracellular vesicle-mediated ferroptosis resistance and immune suppression

Intercellular communication plays a critical role in ferroptosis resistance in GBM, with extracellular vesicles (EVs), particularly exosomes, serving as crucial mediators in maintaining tumor cell iron homeostasis and immune suppression. GBM-derived EVs can remodel the TME, making it more conducive to tumor growth and immune evasion through the delivery of specific signaling molecules (101). Studies have shown that GBM-derived EVs convert AMP to adenosine via the surface molecule CD73+, activating the A2A receptor on T cells and inhibiting their glucose metabolism, leading to immune response paralysis. *In vivo*, defects in exosome synthesis and downregulation of CD73 expression significantly suppressed GBM tumor growth and restored the clonal proliferation of T cells in central and peripheral regions (102). Hong et al. reported that miR-124 in EVs influences the metabolic state of immune cells by regulating the STAT3 signaling pathway. Downregulation of STAT3 expression inhibits M2 microglial polarization, promoting the formation of an immunosuppressive phenotype (103). Moreover, GBM-derived exosomes regulate microglial polarization toward the M2 phenotype via the RAC1/AKT/NRF2 pathway (104). Additionally, these EVs can directly induce ferroptosis in immune cells. Yang et al. reported that GBM-derived exosomes induce lipid accumulation and GPX4 upregulation in dendritic cells (DCs), suppressing their antigen-presenting function and enhancing immune suppression (91). This process not only aids tumor cells in resisting ferroptosis but also inhibits immune cell function by altering iron metabolism within the TME.

Overall, GBM cells form a multilayered ferroptosis resistance network through EV-mediated iron efflux, antioxidant signaling transfer, and immune regulation. This intercellular communication mechanism not only reflects the tumor survival strategy in adapting to iron metabolism stress but also reveals the metabolic basis for the establishment of an immunosuppressive microenvironment.

#### 3.2.2 Metabolic competition and nutrient deprivation

The rapid growth of GBM leads to intense competition for nutrients and oxygen in its microenvironment. Tumor cells meet their energy demands by upregulating metabolic pathways (such as glutamine metabolism and glycolysis) while depriving immune cells of nutrient supply, further suppressing immune responses and significantly affecting ferroptosis sensitivity (105). In this process, the dynamic balance of amino acid, iron, lactate, and lipid metabolism constitutes the key metabolic foundation for immune tolerance and ferroptosis resistance in GBM.

In the TME, tumor cells and T cells compete for cystine uptake. Studies have shown that tumor cells preferentially take up cystine through high expression of SLC7A11, leading to an insufficient supply of cystine for T cells in the TME, thereby inhibiting GSH synthesis and inducing functional exhaustion and promoting ferroptosis in T cells. Cystine deficiency also increases lipid uptake mediated by CD36, promoting lipid peroxidation and ferroptosis in T cells. This process further weakens the antitumor function of T cells, making them more prone to ferroptosis (105). In GBM cells, after SIRT3 is knocked down, mitophagy is upregulated, and the intracellular infusion of GSH leads to the downregulation of the expression of SLC7A11, a key antagonist of ferroptosis. By knocking down SIRT3 and forcing the expression of SLC7A11 in GBM cells, cystine uptake is restored, thereby restoring cellular GSH levels (67). γ-Glutamyltransferase 1 (GGT1) can resist ferroptosis caused by cystine deprivation. Through preclinical studies, Hayashima et al. reported that pharmacological inhibition or knockdown of GGT1 significantly reduces intracellular GSH levels and decreases cell survival under cystine-deprived conditions, leading to GSH depletion and ferroptosis (106). However, exogenous expression of GGT1 can inhibit GSH depletion in GGT1-deficient cells, thereby preventing the accumulation of lipid peroxidation and ferroptosis due to cystine deficiency. Additionally, immunotherapy activates CD8+ T cells to release IFN-γ, downregulates the expression of SLC3A2 and SLC7A11 in tumor cells, inhibits cystine uptake, and thereby promotes ferroptosis in tumor cells (107).

Lactate is the end product of tumor cell glycolysis, and its accumulation in the TIME not only promotes tumor cell survival and invasion but also creates an immunosuppressive environment by inhibiting immune cell functions, such as those of T cells and NK cells. Therefore, targeting lactate metabolism has become crucial for reversing immunosuppression and enhancing immunotherapy efficacy. Research by Wang et al. revealed that lactate can promote CD47-dependent immune evasion through histone lactylation reprogramming of GBM metabolism regulated by the heterochromatin component chromobox protein homolog 3 (CBX3), and additionally, histone lactylation can affect the immune microenvironment in GBM via interferon-regulated transcriptional programs (108). Furthermore, studies have revealed that lactate-driven histone lactylation in GBM cells promotes the expression of IL-10, which is essential for suppressing T-cell activity (26). Li et al. reported that inhibiting the expression of glucose transporter 1 (GLUT1) helps prevent lactate excretion during tumor glycolysis, thereby reducing the number of immunosuppressive tumor-associated TAMs and Tregs (109). Additionally, Zhi et al. reported that lactate accumulation inhibits glycolysis and reduces ATP levels, thereby suppressing the activity of the copper transporter ATP7B and enhancing cuproptosis; moreover, lactate-induced intracellular acidification promotes ferritin (FTH1) dissociation, resulting in the release of endogenous iron, which subsequently triggers ferroptosis (110). These findings suggest that lactate metabolism is not only a key metabolic node in ferroptosis resistance but also a targetable immunometabolic therapeutic pathway.

Iron, as the core element of ferroptosis, is precisely regulated in the TME. Liu et al. reported that GBM patients with high ferroptosis scores were enriched in immune cells, but most were immunoregulatory cells, leading to the formation of an immunosuppressive microenvironment in GBM patients. Additionally, patients with enhanced ferroptosis are characterized by TAM enrichment and M2 polarization (80). Fatty acid-binding protein 7 (Fabp7) is a lipid chaperone protein involved in fatty acid uptake, transport, and metabolism. Freitas-Cortez et al. reported that Fabp7 promotes the accumulation of monounsaturated fatty acids (MUFAs) and triglycerides, inhibits lipid peroxidation and ROS generation to protect GBM cells, and suppresses ferroptosis by upregulating genes such as Bmal1. Increased expression of Fabp7 in CD8^+^ T cells disrupts circadian rhythm genes, induces T-apoptosis, and weakens antitumor immunity; furthermore, Fabp7 confers cancer cell resistance to immunotherapy through the regulation of lipid metabolism and mitochondrial function (111). Nutrient deprivation is a prominent feature of the TIME and significantly influences cancer cell biological behaviors, particularly resistance to ferroptosis. TAR DNA-binding protein-43 (TDP-43), a DNA/RNA-binding protein, was found to be upregulated in GBM under nutrient-deprived conditions through mechanisms that evade the ubiquitin-dependent proteasomal degradation pathway. This upregulation activates autophagy, thereby protecting GBM cells from nutrient deprivation (112).

In summary, the metabolic microenvironment of GBM provides an ecosystem in which ferroptosis resistance and immunosuppression coexist through multidimensional nutrient competition and metabolic reprogramming. Amino acid and lactate metabolism provides continuous antioxidant resources for tumor cells, while the synergistic regulation of lipid and iron metabolism further enhances this metabolic tolerance.

#### 3.2.3 Synergistic effects of key signaling pathways

**Figure 4:**
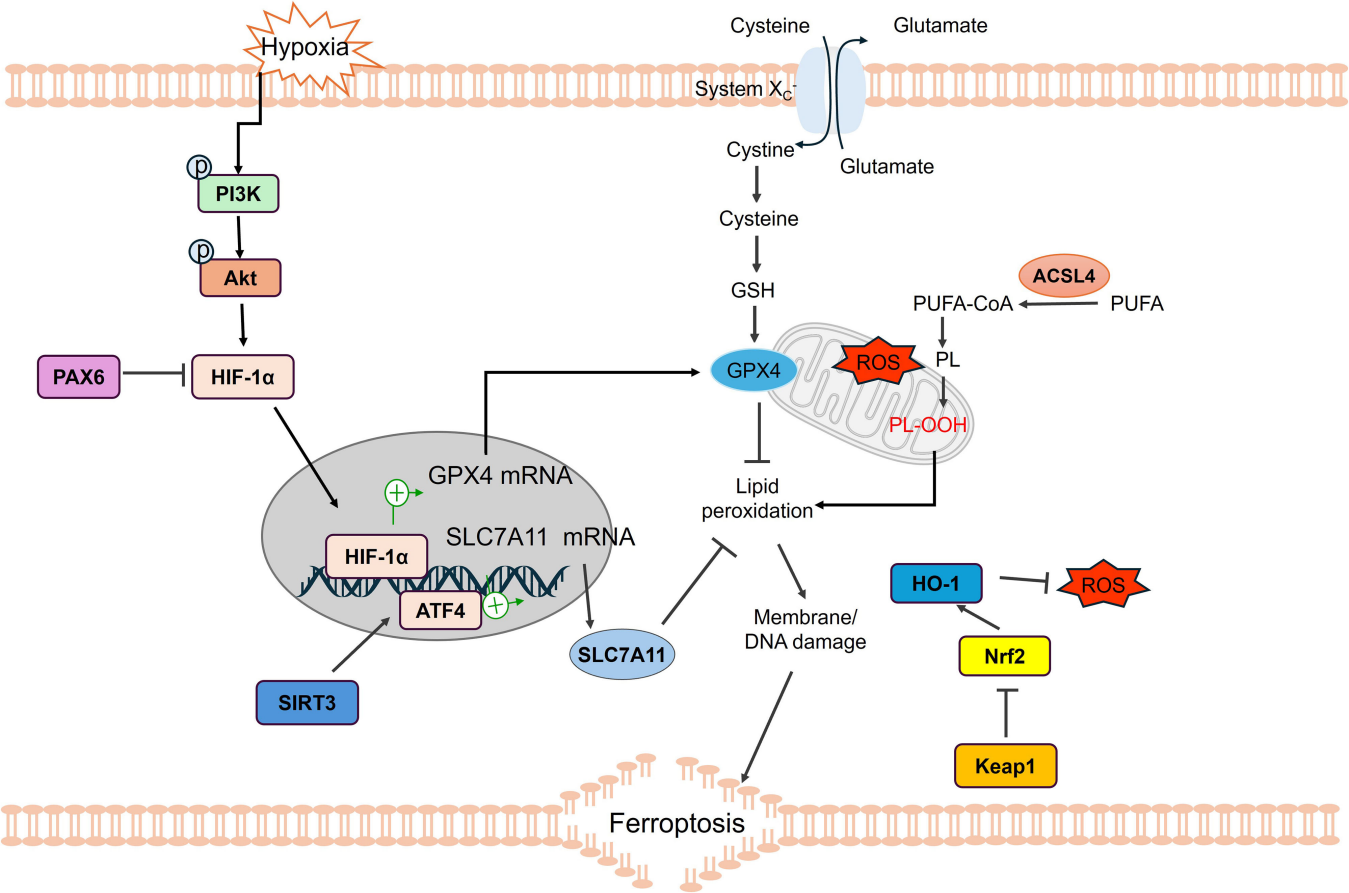
Ferroptosis-related signaling pathways in the TIME of GBM. Ferroptosis in GBM cells is primarily promoted through multiple signaling pathways. Hypoxia-induced PI3K/AKT pathway activation promotes HIF-1α expression. HIF-1α enhances GPX4 and SLC7A11 mRNA levels, thus preventing lipid peroxidation. SIRT3 represses ROS generation and inhibits ferroptosis by enhancing SLC7A11 expression through ATF4 activation. ATF4, Activating Transcription Factor 4; AKT, Protein kinase B; GPX4, Glutathione peroxidase 4; GSH, Glutathione; HO-1, Heme oxygenase 1; NRF2, Nuclear factor erythroid 2-related factor 2; PI3K, Phosphatidyqinositol-3 kinase; ROS, Reactive Oxygen Species; SLC7A11, Solute Carrier Family 7 Member 11; SIRT3, Silent mating type information regulation2 homolog-3; TP53, Tumor protein 53. (Created by Figdraw).

The induction and resistance of ferroptosis in GBM are regulated by the synergistic interaction of multiple signaling pathways, including hypoxia-inducible factor-1α (HIF-1α), Nrf2, heme oxygenase-1 (HO-1/HMOX1), signal transducer and activator of transcription (STAT1), p53, and Sirtuin 3 (Sirt3) (**Figure 4**). These pathways not only determine tumor cell tolerance to oxidative stress but also shape the suppressive TIME through metabolic and immune signaling interactions.

The HIF-1α signaling pathway is highly active in the hypoxic microenvironment of GBM and is involved in the regulation of ferroptosis. HIF-1α, the main subunit of HIF, is stably expressed under hypoxic conditions. Wang et al. reported that HIF-1α can regulate the malignant progression of GBM under hypoxic conditions and that hyperbaric oxygen significantly inhibits HIF-1α expression, increasing the sensitivity of GBM cells to chemotherapy (113). Luo et al. found that paired-box gene 6 (PAX6) had reduced expression in two glioma cells (U251 and U373). PAX6 overexpression downregulated HIF-1α and elevated ROS levels, thereby promoting ferroptosis by repressing GPX4 and GSH (114). LncRNA LINC01956 is regulated by HIF-1α under hypoxic conditions in GBM. Its overexpression accelerates GBM progression and induces TAM polarization toward the M2 phenotype. Targeting HIF-1α reduces LINC01956 expression, thereby inhibiting GBM progression (115).

TGF-β1 induces HIF-1α overexpression and nuclear accumulation by upregulating NOX4 expression and ROS production, ultimately leading to metabolic reprogramming and promoting EMT. This process relies on the Smad signaling pathway and can be blocked by inhibiting the PI3K/AKT/HIF-1α pathway to disrupt TGF-β1-induced metabolic reprogramming and EMT (116). TGF-β1-induced metabolic reprogramming is closely linked to immunosuppression in the TME, potentially affecting immune cell activity by modulating tumor cell metabolism. Sun et al. reported that HIF-1α upregulates SLC7A11 expression via the PI3K/AKT pathway under hypoxia, inhibiting lipid peroxidation and thereby resistance to ferroptosis. Inhibiting AKT or HIF-1α expression significantly reversed the upregulation of SLC7A11 expression, restoring cellular sensitivity to ferroptosis. Hypoxia markedly decreases sulfasalazine-induced ferroptosis sensitivity, suggesting that HIF-1α-mediated antioxidant metabolism is a critical survival mechanism in GBM (117). Roxadustat, a prolyl hydroxylase (PHD) inhibitor, stabilizes and activates HIF-α by inhibiting PHD activity. Preclinical studies revealed that roxadustat-treated GBM cells exhibit significantly increased lipid peroxidation and intracellular iron accumulation. Induced ferroptosis is associated with HIF-2α upregulation, which further increases the expression of lipid-regulating genes and promotes lipid peroxidation (118). Additionally, roxadustat-induced ferroptosis is accompanied by the release of immunogenic signals, potentially amplifying antitumor immune responses. Notably, HIF-2α plays a unique role in regulating immune cell infiltration. Espinoza et al. reported in preclinical studies that the HIF-2α inhibitor PT2385 alleviates the immunosuppressive TIME and synergizes with ICB therapies (e.g., αPD-1 and αTIM-3 antibodies) to promote long-term tumor survival. The results of this study revealed that combining PT2385 with ICB reduces the infiltration of macrophages and Tregs while shifting the transcriptional profile of these TAMs toward an antitumor phenotype. *In vitro* experiments further demonstrated that HIF-2α deficiency impedes microglial polarization toward the protumor M2-like phenotype and suppresses Treg migration (119).

Nrf2 is a key factor that regulates ferroptosis tolerance, and its abnormal activation is closely related to the survival, proliferation, and therapeutic resistance of tumor cells. Its overexpression promotes the proliferation and malignant transformation of GBM cells while inhibiting the occurrence of ferroptosis (120). The upregulation of Nrf2 not only affects ferroptosis but also promotes the recruitment of TAMs and M2 polarization by upregulating ANXA1 protein expression, thereby leading to the formation of an immunosuppressive microenvironment. Li et al. confirmed that the overexpression of CircMAN1A2 can reduce the expression of Nrf2 and ANXA1 and inhibit M2 polarization of TAMs, thereby reversing immunosuppression. Additionally, it can directly bind to the TEP1 protein, disrupt the interaction between TEP1 and KEAP1, promote the degradation of Nrf2, and increase sensitivity to ferroptosis, thus inhibiting the progression of GBM (121). Src tyrosine kinase, a critical signaling molecule, is closely associated with tumor progression and therapeutic resistance. Cirotti et al. reported that Src tyrosine kinase stabilizes and activates the Nrf2 pathway, promoting GBM resistance to ferroptosis (122). In IDH-mutant gliomas, the PI3K/AKT signaling pathway is significantly activated and suppresses ferroptosis by regulating the expression and activity of Nrf2 (123). Moreover, AKT stabilizes Nrf2 by inhibiting GSK3β activity, thereby enhancing its antioxidant and antiferroptotic functions. ABCC1 (also known as MRP1) is an ATP-binding cassette (ABC) transporter protein capable of exporting GSH from cells. Nrf2 increases the sensitivity of cells to ferroptosis by upregulating ABCC1 expression, promoting GSH efflux, and leading to a decrease in intracellular GSH levels (124). Interleukin-4-induced protein 1 (IL4I1) is a newly defined tryptophan-metabolizing enzyme that is upregulated in GBM patients and is associated with poor prognosis. A preclinical study performed by Xu and colleagues reported that IL4I1 catalyzes tryptophan metabolism to produce indole-3-pyruvate (I3P), which can scavenge free radicals and directly bind to Nrf2 to reduce its ubiquitination level, thereby stabilizing Nrf2 expression. Further experiments revealed that knocking down Nrf2 expression significantly attenuated the antiferroptotic effect induced by IL4I1, while inhibiting the activity of IL4I1 or its downstream signaling pathways may restore ferroptosis sensitivity and enhance antitumor immune responses (125).

HO-1 plays a crucial role in the regulation of ferroptosis. High expression of HO-1 inhibits ferroptosis by reducing lipid peroxidation and ROS production. For example, HMOX1 is transcriptionally activated by IFI16, significantly decreasing radiation-induced lipid peroxidation, ROS generation, and intracellular Fe^2+^ content, thereby suppressing ferroptosis and enhancing radioresistance (126). Studies have shown that the expression of HO-1 is closely related to the immunosuppressive activity of TAMs. Inhibiting HO-1 expression can significantly reduce the immunosuppressive function of TAMs, decrease IL-10 release, downregulate STAT3 activation and PD-L1 expression, and activate T cells, all of which affect PD-L1 and IDO1 expression. Further research has indicated that inhibiting HO-1 also reduces the enzymatic activity of IDO1 and the gene expression of ARG-2, reversing the immunosuppressive function of TAMs and enhancing ferroptosis (127). Notably, the expression of HO-1 is regulated by Nrf2, and the nuclear translocation of Nrf2 is inhibited by KEAP1. For instance, the natural compound cryptotanshinone significantly induces ferroptosis in GBM cells by activating the KEAP1/Nrf2/HMOX1 signaling pathway (128). Betulinic acid (BA), a natural pentacyclic triterpenoid, reduces cell viability by inhibiting the PI3K/Akt pathway while activating the Nrf2/HO-1 pathway, increasing oxidative stress and ferroptosis (129). These findings suggest that the Nrf2/HO-1 signaling pathway can influence the activity and function of immune cells by modulating the redox balance in the TME. Targeting this axis may be an effective strategy for reshaping the immune microenvironment.

STAT3 and STAT1 are two critical signaling molecules in the immune microenvironment of GBM that play significant roles in the regulation of ferroptosis. In a mouse model, Sun et al. reported that SLC10A3 affects the expression of GPX4 by regulating the transcription and phosphorylation of STAT3, thereby controlling the ferroptosis process and tumor cell survival in GBM (130). The STAT3 signaling pathway not only directly regulates the biological behaviors of tumor cells but also promotes immune escape by influencing the function of immune cells in the TIME. For instance, the activation of STAT3 leads to T-cell exhaustion, suppressing T-cell antitumor activity. Simultaneously, STAT3 modulates the secretory function of TAMs, facilitating tumor angiogenesis and the release of immunosuppressive cytokines such as IL-10 and TGF-β (131). ARPC1B (actin-related protein 2/3 complex subunit 1B) is significantly upregulated in GBM. It inhibits the ubiquitination and degradation of STAT1 by preventing the binding of the E3 ubiquitin ligase NEDD4L to STAT1 and promoting the interaction between STAT1 and the deubiquitinating enzyme USP7, thereby increasing its stability. Stabilized STAT1 induces the production of IL-10 (an immunosuppressive cytokine), promotes the “M2” polarization of macrophages, and weakens the efficacy of ICB, leading to immune resistance (132). DDX58 (also known as RIG-I) is a pattern recognition receptor that is involved primarily in recognizing viral RNA and activating innate immune responses. Wang et al. further reported that radiation-induced senescent GBM cells inhibit TRIM21-mediated ubiquitination of STAT1 via the regulation of its stability by DDX58. Activated STAT1 increases CSF1 transcription, promoting TAM recruitment and M2 polarization (108). The immunosuppressive function of M2 macrophages aids tumor immune evasion and progression, indirectly reducing ferroptosis sensitivity.

p53, as a key tumor suppressor, not only participates in DNA damage repair and cell cycle regulation but also influences tumor cell metabolism and oxidative stress responses by regulating genes such as TIGAR. In GBM, FRGs such as STEAP3, HSPB1, and MAP1LC3A affect tumor cell fate by modulating processes such as the p53 signaling pathway, senescence, and autophagy. Studies have shown that key ferroptosis-related genes are strongly correlated with immune-related factors and significantly contribute to immune cell infiltration and the expression of immunomodulatory factors (34). Ferroptosis may influence tumor cell ICD by regulating p53 signaling, thereby activating antitumor immune responses. The p53 signaling pathway has complex functions in GBM, where the loss or mutation of p53 in certain mutant GBMs can lead to iron homeostasis disruption and metabolic remodeling imbalance (46). Cai et al. reported in preclinical studies that in TP53-mutant GBMs, TP63 expression is significantly upregulated, which inhibits ferroptosis by reducing ROS accumulation and lipid peroxidation. Further mechanistic analysis revealed that TP53 mutation activated the Wnt/β-catenin pathway, promoting β-catenin nuclear accumulation and transcriptional upregulation of TP63. TP63 directly increases the expression of the key ferroptosis inhibitor GPX4, resulting in the formation of a TP53 mutation-TP63-GPX4 axis that suppresses ferroptosis (133). Notably, PELATON, also known as the long noncoding RNA LINC01272, can alleviate ferroptosis driven by mutant p53. In p53 wild-type GBM cells, PELATON inhibits p53 expression and regulates BACH1 and CD44 expression, with its function potentially dependent on p53 status. It suppresses ROS production, reduces Fe^2+^ levels, and promotes SLC7A11 expression through mutant p53-mediated ROS-ferroptosis pathways while inhibiting ACSL4 and COX2 expression. Additionally, knocking down PELATON induces mitochondrial changes, enhances sensitivity to ferroptosis inducers, and inhibits GBM cell proliferation and invasion (134).

Sirtuin 3 (SIRT3) is a mitochondrial deacetylase that is highly expressed in GBM and further upregulated during RSL3-induced ferroptosis. Li et al. demonstrated that inhibiting SIRT3 promotes the accumulation of mitochondrial Fe^2+^ and ROS and regulates the expression of SLC7A11, thereby triggering mitophagy and enhancing the sensitivity of GBM cells to ferroptosis. Additionally, SIRT3 can activate the transcription factor ATF4 to regulate the transcription of SLC7A11 (67). In another study, overexpression of SIRT3 was shown to further increase the sensitivity of GBM cells to ferroptosis by activating NCOA4-mediated autophagy (135). Notably, an autophagy-dependent FRG coprediction model suggested that the expression of SIRT1 may be associated with macrophage infiltration and immune tolerance in glioma tissues (136). Fu et al. revealed that the expression of HO-1 is closely linked to the regulation of SIRT1. The inhibition of SIRT1 expression enhances HO-1 expression, and the natural compound polyphyllin I induces ferroptosis by activating the Sirt1/Nrf2/HO-1/GPX4 cascade, thereby suppressing tumor growth (137).

In summary, HIF-1α, Nrf2/HO-1, STAT, p53, and SIRT3 pathways form a complex dynamic regulatory network in GBM. Together, they maintain redox homeostasis, remodel the metabolic environment, and regulate ferroptosis resistance through the TME.

## 4 Clinical translation to target ferroptosis and immune combination strategies

### 4.1 Therapeutic potential and immunomodulatory effects of ferroptosis inducers

Preclinical studies have suggested that ferroptosis inducers, such as erastin, RSL3, and FIN56, have significant antitumor activity in GBM. These agents trigger PCD in tumor cells by disrupting iron homeostasis, inhibiting antioxidant systems, or enhancing lipid peroxidation while also modulating the TIME of GBM.

Erastin, one of the earliest discovered ferroptosis inducers, inhibits system Xc^-^ (SLC7A11/SLC3A2), thereby impairing GSH synthesis, leading to ROS accumulation and inducing GBM cell death (138). PGRMC1 is considered an oncogenic factor, and high expression of PGRMC1 increases the sensitivity of GBM cells to the ferroptosis inducer erastin. Altered expression of PGRMC1 may influence the response of GBM cells to erastin by promoting tumor-associated inflammatory responses and increasing the number of tumor-infiltrating neutrophils (139). LRRK2 (leucine-rich repeat kinase 2) plays a crucial protective role in immune cells, particularly in combating ferroptosis. Treatment with erastin revealed that LRRK2-knockout RAW 264.7 murine macrophages exhibited significantly reduced resistance to ferroptosis, manifested as markedly increased lipid peroxidation and ROS generation and significant decreases in mitochondrial membrane potential and mitochondrial respiratory function (140). SLC39A14, a zinc-iron transporter, primarily facilitates the extracellular-to-intracellular transport of non-transferrin-bound iron (NTBI). Under iron overload conditions, increased SLC39A14 expression may lead to intracellular iron accumulation, thereby triggering ferroptosis. Studies have shown that SLC39A14 knockdown significantly enhances erastin-induced ferroptosis, which is characterized by reduced intracellular MDA and Fe^2+^ levels, increased GSH levels, and suppressed expressions of ferroptosis-related proteins such as GPX4, NRF2, and SLC7A11 (141). Additionally, in preclinical glioma models, SLC39A14 knockdown inhibited tumor cell proliferation, migration, and invasion while promoting erastin-induced ferroptosis, significantly suppressing the growth of xenograft tumors in mice.

RAS-selective lethal 3 (RSL3) is a known ferroptosis inducer. RSL3 directly inhibits GPX4, leading to the accumulation of lipid peroxides and cell membrane damage, thereby inducing ferroptosis (142). xCT is a critical component of System Xc^-^ and is responsible for the uptake of intracellular cystine and the synthesis of glutathione. Its downregulation further exacerbates oxidative stress and ferroptosis. The NF-κB pathway plays a significant role in immune regulation, and its activation may affect the function of TAMs and other immune cells, thereby altering the immune state of TIME. Studies have shown that RSL3 activates the NF-κB pathway, further downregulating the expression of ferroptosis-related proteins such as ATF4 and xCT (143). Additionally, RSL3 promotes ferroptosis by affecting mitochondrial function. Preclinical studies have indicated that RSL3 treatment induces typical ferroptosis-related morphological changes in the mitochondria of GBM cells, including mitochondrial shrinkage, increased mitochondrial membrane density, and reduced mitochondrial cristae (67). Mitochondria are key organelles involved in iron metabolism and oxidative stress, and their dysfunction leads to the accumulation of iron ions and ROS, thereby triggering ferroptosis. Moreover, RSL3 increases the expression of ferroptosis-related proteins such as IRP1 and HO-1. These changes suggest that RSL3 promotes ferroptosis by disrupting mitochondrial structure and function. Furthermore, the ferroptosis inducer arsenic trioxide (ATO) can synergize with photodynamic therapy (PDT) to induce significant antitumor effects in preclinical tumor models (including recurrence model, breast cancer model and patient-derived model). Mechanistically, ATO and PDT combination mediated mitochondrial damage, contributed to ferroptosis activation, and remodeled the TIME (144).

Fer-1 is a specific ferroptosis inhibitor that prevents ferroptosis by inhibiting lipid peroxidation. In GBM cell lines (U87 and U251), BRIP1 downregulation or erastin-treated U251 cells, Fer-1 restored cell viability and reversed the increase in lipid peroxidation and iron accumulation. It can also reverse the negative effects caused by BRIP1 downregulation, leading to a decrease in MDA levels and intracellular Fe^2+^ content and an increase in GSH, Gpx4, and SLC7A11 levels (145). In T98G and U251 cell lines, MXRA8 knockdown inhibited cell growth, increased intracellular Fe^2+^ and MDA levels, and reduced M2-type macrophage infiltration, whereas Fer-1 reversed these conditions (84).

Ferroptosis inducer 56 (FIN56) is a ferroptosis inducer that inhibits the activity of GPX4, thus leading to the accumulation of lipid peroxides within cells and thereby inducing ferroptosis. This mechanism is particularly prominent in tumor cells, as they are generally more sensitive to ferroptosis. Studies have shown that FIN56 can significantly reduce the survival rate of GBM cells (such as LN229 and U118 cells) by inducing ferroptosis, increasing lysosomal membrane permeability, inhibiting cell proliferation, and causing cell cycle arrest. In a subcutaneous nude mice model, FIN56 treatment also dampened the growth of tumor cells by inducing ferroptosis (146). In the IR-Surv GBM model, FIN56 and RSL3, as ferroptosis inducers, suppress GPX4 activity, induce lipid peroxidation, and trigger cell death (147). Additionally, the combination of FIN56 with photothermal therapy (e.g., the Graphdiyne-FIN56-RAP nanoplatform) enhances therapeutic efficacy, enabling penetration of the blood–brain barrier (BBB) and the release of FIN56 within the TIME to further amplify ferroptosis induction (148). Collectively, those preclinical studies suggest that FIN56, when combined with other antitumor agents/therapies, exhibits enhanced antitumor effects in GBM.

### 4.2 Enhancing the efficacy of temozolomide and sensitivity to radiotherapy

TMZ primarily induces cell damage through DNA methylation, but resistant cells often evade treatment by activating repair systems and metabolic reprogramming. TMZ resistance is among the main reasons for the failure of GBM treatment. Accumulated preclinical studies have verified that ferroptosis inducers can overcome this challenge through multiple pathways. Yin et al. constructed a metal–phenolic network (TBFP-MT MPN) system based on PEG-polyphenol, which reduces drug efflux by inhibiting mesenchymal–epithelial transition signaling and blocking pyrimidine synthesis through the inhibition of dihydroorotate dehydrogenase (DHODH), thereby lowering the levels of the DNA repair protein O6-methylguanine-DNA methyltransferase (MGMT) and enhancing TMZ cytotoxicity (149). Preclinical studies suggested that this system can induce ferroptosis by disrupting the DHODH/GPX4 defense system, with the ability to inhibit the growth of resistant tumors and prolong the survival of animal tumor models. The combination of ferroptosis inducers with TMZ produces synergistic killing effects, and GPX4 inhibitors such as RSL3 can significantly amplify the cytotoxic effects of TMZ. Yang et al. reported that the combined use of RSL3 and TMZ significantly inhibited the growth of glioma cells and reduced their invasive ability. This synergistic effect was observed similarly in both IDH1-mutant and wild-type glioma cells, suggesting that RSL3 may enhance TMZ efficacy by inducing ferroptosis. TMZ sensitivity is negatively correlated with the expression of ferritin (FTH1 and FTL), and high ferritin expression may lead to TMZ resistance through the inhibition of ferroptosis (150). Studies have shown that the AKT inhibitor ipatasertib (Ipa) combined with TMZ, can significantly increase ferroptosis in IDH-mutant glioma cells (123). This synergistic effect is achieved by inhibiting AKT activity, further reducing Nrf2 stability, and activating ferroptosis-related pathways in cells. In preclinical animal models, this combination therapy significantly prolonged survival.

Boric acid enhances the chemosensitivity of TMZ-resistant GBM cells by activating the ferritin autophagy pathway and disrupting cellular iron homeostasis. In TMZ-resistant GBM cells (such as A172-R and T98G-R cells), boric acid treatment significantly increased the expression level of NCOA4, elevated intracellular iron levels, and triggered lipid peroxidation and GSH depletion, thereby promoting ferritin autophagy and inducing ferroptosis (151). In TMZ-resistant GBM cells, high expression of NRF2 mediates GSH efflux through ABCC1, leading to a significant decrease in GSH levels when System Xc^-^ is blocked (erastin treatment), thereby inducing ferroptosis (124). Haloperidol, a DRD2 antagonist, significantly induces ferroptosis in GBM cells by inhibiting DRD2 receptor activity. A preclinical study indicated that haloperidol triggers endoplasmic reticulum stress by inhibiting the effect of TMZ on the cAMP/PKA signaling pathway, subsequently inducing autophagy. This autophagy further mediates the downregulation of FTH1 expression, leading to ferroptosis. The combination of haloperidol and TMZ effectively enhances the efficacy of TMZ and suppresses adaptive resistance in GBM cells (152). These results demonstrate that drug combinations targeting iron homeostasis disruption, GPX4 inhibition, and stress response induction can effectively reverse TMZ resistance.

Radiation therapy can induce lipid peroxidation by generating reactive oxygen species (ROS), which are key triggers of ferroptosis. In GBM, CA9 (carbonic anhydrase 9), a downstream target gene of hypoxia-inducible factor HIF-1α, has been shown to increase radiation-induced ferroptosis when it is knocked down, leading to a significant increase in intracellular ROS levels and increasing tumor cell sensitivity to radiation-induced oxidative stress (153). Studies indicate that carbon ions combined with photon radiation therapy induce ferroptosis in GBM through the NCOA4-mediated ferritinophagy pathway. The clustered DNA double-strand breaks induced by combined irradiation result in the accumulation of double-stranded DNA fragments in the cytoplasm, activating the cGAS-STING-mediated cytosolic DNA sensing pathway, which further promotes NCOA4-FTH1 axis-driven ferritinophagy, ultimately leading to iron-dependent cell death (154). These findings suggest that targeting hypoxia-adaptive metabolic pathways and ferroptosis signaling can synergistically increase the efficacy of radiotherapy. However, some tumor cells can acquire radioresistance by upregulating the expression of genes that regulate antioxidant enzymes and iron metabolism. Zhou et al. reported that the senescence-associated gene IFI16 promotes radioresistance by activating the HMOX1 pathway, which clears intracellular free iron and suppresses lipid peroxidation. Researchers have discovered that the diabetic drug glibenclamide specifically targets the pyrin domain of IFI16. In animal models, combining this drug with radiotherapy extended the survival of mice with orthotopic transplanted tumors to 60 days (compared with 28 days in the control group) and reduced the tumor volume by 75% (126). HMOX1 inhibitors targeting this mechanism hold promise for overcoming the ferroptosis inhibition barrier and restoring radiosensitivity.

### 4.3 Ferroptosis-targeting nanomaterials

**Figure 5:**
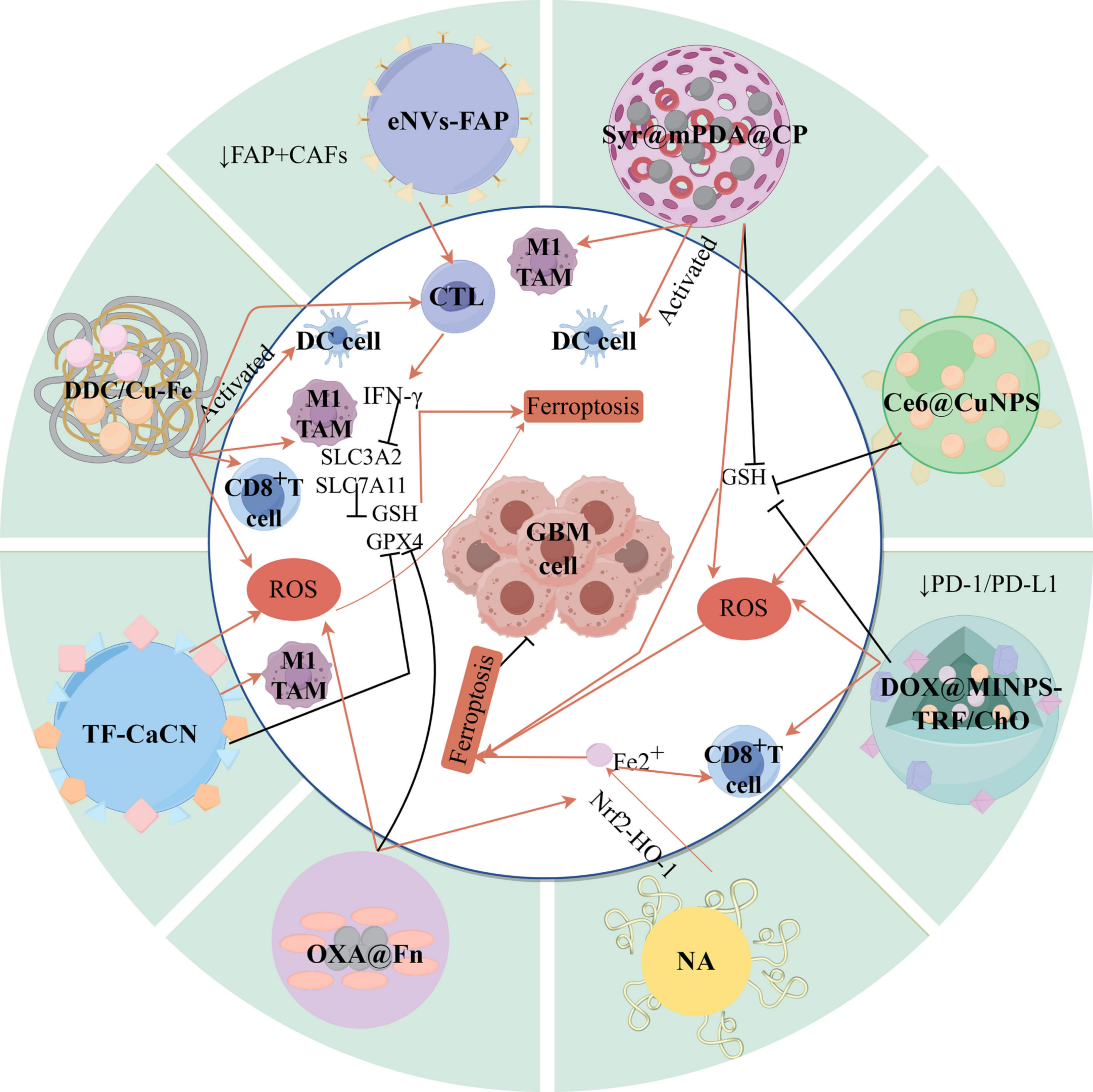
Ferroptosis-targeting nanomaterials in GBM therapy by mediating TME. Multiple nanomaterials have been developed for treating GBM through targeting ferroptosis and modulating the tumor microenvironment (TME). DDC/Cu-Fe activates dendritic cells (DCs), promotes M2-type tumor-associated macrophages (TAMs) to polarize toward M1-type TAMs, activates CD8^+^T cells, and inhibits GPX4 activity by stimulating CTLs to secrete IFN-γ, thereby suppressing SLC3A2 and SLC7A11. This process depletes GSH to promote ferroptosis and additionally enhances reactive oxygen species (ROS) production. TF-CaCN promotes ferroptosis by enhancing ROS production and facilitates M1 TAM polarization. OXA@Fn stimulates Fe²^+^ and ROS generation while inhibiting GPX4 activity, depleting GSH to induce ferroptosis. NA promotes Fe^2+^ production via the Nrf2-HO-1 pathway, thereby activating CD8+ T cells and triggering ferroptosis. DOX@MINPS-TRF/ChO promotes ferroptosis by enhancing ROS production and depleting GSH, while stimulating CD8+ T cell activation and downregulating PD-1/PD-L1 expression. Ce6@CuNPS promotes ferroptosis by enhancing ROS production and depleting GSH. Syr@mPDA@CP promotes M1-type TAM polarization and activates DCs, enhancing ROS production and depleting GSH to induce ferroptosis. eNVs-FAP stimulates CTL secretion of IFN-γ, inhibits GPX4 activity by suppressing SLC3A2 and SLC7A11, depletes GSH to trigger ferroptosis, and additionally downregulates FAP+CAFs. CAFs, Cancer associated fibroblasts; CTL, Cytotoxic T Lymphocyte; DC, Dendritic Cell; FAP, Fibroblast activation protein alpha Gene; GPX4, Glutathione peroxidase 4; GSH, Glutathione; IFN-γ, Interferon-gamma; PD-1, Programmed death 1; PD-L1, Programmed Cell Death Ligand 1; ROS, Reactive Oxygen Species; SLC3A2, Solute Carrier Family 3 Member 2; SLC7A11, Solute Carrier Family 7 Member 11; TAM, Tumor-associated macrophage. (Created by Figdraw).

In recent years, the use of nanomaterials has provided new insights into the clinical translation of ferroptosis-targeted therapy for GBM. Traditional ferroptosis drugs face challenges such as poor BBB permeability, high systemic toxicity, and insufficient tumor targeting. Nanodelivery systems, through surface modification, biomimetic encapsulation, and responsive design, enable controlled drug release and tumor-specific accumulation, thereby enhancing therapeutic efficacy and reducing off-target tissue damage. The application of nanotechnology in GBM ferroptosis therapy is advancing toward precise delivery and immune synergy. By employing biomimetic modifications, metal regulation, and intelligent responsive designs, nanocarriers not only overcome BBB limitations but also achieve spatiotemporal codelivery of ferroptosis inducers and immune modulators. Increasing preclinical studies have confirmed the antitumor effects of nanomaterials in GBM (**Table 1****) (****Figure 5**).

**Table 1:** Nanomaterials for GBM therapy that target ferroptosis.

Nanoplatform type	Objective	Mechanism of action	Immunomodulatory effects	Key findings	Ref.
Epr@DNA-Octa	Combination of focused ultrasound + microbubbles to temporarily, noninvasively, and repeatedly open localized regions of the BBB	Loaded with epirubicin	Not explicitly reported	50% increase in median survival and 73% extension in maximum survival in intracranial GBM models	(155)
DOX@MINPs-TRF/ChO	Enhanced Trf-mediated BBB penetration + GSH depletion	Cu^2+^Induction of copper reduction synergized with chemotherapy	Significant increase in maturation rate of bone marrow-derived dendritic cells, notable rise in tumor-infiltrating CD8^+^T-cell proportion, downregulation of PD-1/PD-L1 expression	Achieved synergistic cuproptosis/ICB/ICD, reprogrammed tumor microenvironment, and enhanced anti-tumor efficacy	(156)
	Enhances BBB permeability, improves the enzymatic resistance of siHDGF	Controls the release of TMZ and siHDGF	Not explicitly reported	Significantly inhibits tumor growth and prolongs survival	(157)
Ce6@Cu NPs	Promotes the combination of sonodynamic-triggered cuproptosis and ferroptosis	Ultrasound-triggered lipid peroxidation + Cu ^2+^ Induces copper reduction	Not explicitly reported	Inhibits tumor growth with minimal side effects	(158)
Syr@mPDA@CP	MCT4 inhibitor blocks lactate efflux	Inhibits lactate outflow, lactate accumulation promotes Fe ^2+^ release + ATP7B inactivation	Promotes T lymphocyte differentiation and infiltration, transforms M2-type TAMs into M1-type TAMs, significantly increases DC cell maturation rate, significantly reduces Treg and MDSC cells, reverses the immunosuppressive environment	Establishes a “lactate metabolism-metal ion death axis, “ effectively enhances tumor cell cuproptosis and ferroptosis, increases immunogenicity, and activates potent anti-tumor immunity	(110)

This table summarizes the key characteristics of various nanoplatforms for ferroptosis therapy in GBM, including their targeting strategies, ferroptosis induction mechanisms, immunomodulatory effects, and main experimental findings. Nanotechnology enhances ferroptosis induction efficiency and improves the tumor immune microenvironment through multiple synergistic mechanisms, providing promising strategies for GBM treatment. GBM, glioblastoma; BBB, blood–brain barrier; TfR1, transferrin receptor 1; STING, stimulator of interferon genes; OMVs, outer membrane vesicles; siRNA, small interfering RNA; TAMs, tumor-associated macrophages; DC, dendritic cells; TRF, transferrin; PD-1, programmed cell death protein 1; PD-L1, programmed death-ligand 1; ICD, immunogenic cell death; TMZ, temozolomide; siHDGF, siRNA targeting hepatoma-derived growth factor; NPs, nanoparticles; MCT4, monocarboxylate transporter 4; CAT, catalase; POD, peroxidase; GPx, glutathione peroxidase; GSH, glutathione; ·OH, hydroxyl radical.

Hu et al. developed fibroblast activation protein α (FAP) gene-engineered tumor cell-derived exosome-like nanovesicles (eNVs-FAP) and reported that eNVs-FAP can induce a strong CTL immune response, release IFN-γ, and deplete FAP+ cancer-associated fibroblasts (CAFs). By clearing FAP+ CAFs, eNV-FAP significantly reduces the number of immunosuppressive cells (such as M2-type TAMs, MDSCs, and Tregs) in the TME, thereby reversing the immunosuppressive state and enhancing antitumor immune responses (159). Zhang et al. constructed homologous magnetic-targeted immune vesicles (Sp-Exos/AIs), further confirming that activating ferroptosis can enhance the immune amplification effect of photodynamic therapy, promoting the function of tumor-specific T lymphocytes and the phenotypic transformation of macrophages (144). Additionally, Tian et al. demonstrated that modifying the surface of EVs to carry brain tumor-targeting peptides (e.g., RGD peptide) combined with short-pulse radiotherapy significantly improved the targeting efficiency of EVs in GBM. Further studies revealed that EVs loaded with PD-L1 siRNA can reverse radiotherapy-induced PD-L1 expression, recruit tumor-associated myeloid cells, increase CD8^+^ T-cell activity, inhibit tumor growth, and prolong animal survival (160). Cao et al. developed a nanomaterial (NA) composed of gold clusters and optimized GBM-targeting peptides, which can efficiently target GBM cells. Research has shown that NA induces ferroptosis by regulating the Nrf2/HO-1 signaling pathway, where increased HO-1 activity leads to elevated intracellular iron levels, triggering ferroptosis. Furthermore, NA treatment significantly increased the infiltration of tumor-killing lymphocytes within tumors, suggesting its potential to enhance antitumor effects by activating the immune system (161). Tushe et al. developed a water-in-oil nanoemulsion loaded with ZnPPIX (NE-ZnPPIX), which effectively reduced the immunosuppressive activity of *in vitro*-derived TAMs by decreasing CD163 expression (162). Wang et al. developed a complex (DDC/Cu-Fe) formed by chelating diethyldithiocarbamate (DDC) with copper and ferrous ions, which activates T-cell immune responses in the TME, inhibits the ability of TAMs to alleviate immunosuppression, and can be encapsulated in nanomaterials to target the BBB and overexpress nutrient transporters in the GBM for anticancer immunity (163). Jin et al. developed a calcium-based nanozyme (CaCN) with peroxidase-like (POD) activity, which disrupts mitochondrial morphology, induces calcium overload leading to GBM apoptosis, triggers intracellular ferroptosis, and remodels the immune microenvironment of GBM (164). Xue et al. loaded oxaliplatin (OXA) into ferritin to prepare the glioma-targeting inclusion complex OXA@Fn, which effectively penetrates the BBB and enhances iron death-induced T-cell-mediated antitumor immune responses *in vivo* (165).

In addition to the construction of nanomaterials, a significant amount of research has focused on the development of nanoplatforms to target GBM and enhance immune responses for therapeutic effects, as shown in **Table 1**. These innovative approaches provide new directions for overcoming therapeutic resistance in GBM.

The interactive activation strategy between cuproptosis and ferroptosis offers novel therapeutic targets for regulating tumor metabolism and the TIME. However, these systems still require further validation in terms of pharmacokinetic safety and clinical feasibility to advance ferroptosis-directed immunotherapy from the laboratory to clinical practice.

### 4.4 Immunotherapy combined strategies

Ferroptosis induction reshapes the suppressive TIME of GBM through multiple mechanisms. Following the induction of ferroptosis, the accumulation of lipid peroxides can downregulate the expression of immune checkpoint molecules. Through cholesterol depletion, the DOX@MINP-TRF/ChO drug-loaded nanosystem downregulates the expression of PD-1 and PD-L1, significantly increasing the proportion of infiltrating CD8^+^ T cells in GL261 glioma-bearing mice (156). This system enhances the ICD effect and maintains the ICB effect.

Immune checkpoint inhibitors (such as PD-1/PD-L1 inhibitors) have limited efficacy in GBM treatment, primarily because of the highly suppressive TIME. Preclinical studies have indicated that ferroptosis can convert protumor M2 TAMs into M1 TAMs, reducing the formation of a suppressive TIME (100). For example, targeting integrin α5 (ITGA5) can disrupt signaling between tumor cells and exosomes, promoting the conversion of M2 macrophages to M1 macrophages, enhancing T-cell-mediated tumor killing, and improving anti-PD-1 treatment responses (166). In tumor metabolic regulation, a nanoscale system targeting lactate metabolism inhibits MCT4 function, leading to intracellular lactate accumulation. This not only synergistically activates ferroptosis and cuproptosis but also significantly increases CD8^+^ T-cell infiltration, reduces the proportion of M2 macrophages, and effectively reverses immunosuppression (110). Li et al. developed NO/ROS-scavenging nanoparticles (NPs) that target NO and ROS released by tumor-associated myeloid cells (TAMCs), protecting T cells from exhaustion and significantly enhancing the efficacy of anti-PD-1 antibodies in GBM models (167). Similarly, a photodynamic immunotherapy platform constructed from amphiphilic photosensitizers (PSs) and immune checkpoint inhibitors (atezolizumab) successfully promoted cytotoxic T-cell activation in GBM cell lines and 3D spheroid models, enhancing the efficacy of immune checkpoint inhibitors. Photodynamic therapy (PDT) increases blood–brain barrier permeability, promotes immune cell infiltration, and reprograms or inactivates immunosuppressive TAMs, whereas atezolizumab blocks the PD-L1/PD-1 pathway, further increasing T-cell antitumor activity (168).

Several preclinical investigations have shown that nanocarriers and immunotherapy combination can enhance tumor targeting and overcome the BBB limitation. For example, RSL3-loaded NK cell–exosome hybrid nanovesicles (hNRVs) can specifically recognize GBM cells, induce ferroptosis, promote NK cell recruitment, and release FASL and IFN-γ into the TME. FASL can effectively lyse tumor cells, while IFN-γ stimulates dendritic cell maturation and enhances immune responses (169). Liu et al. developed a nanodelivery system (TMZ/siPD-L1@GLPN/dsb) capable of simultaneously delivering TMZ and siPD-L1, reducing MGMT protein expression, and significantly increasing the sensitivity of TMZ-resistant GBM cells to TMZ. This synergistic effect markedly reversed the immunosuppressive TIME, decreasing the ratio of CD4+CD25+FoxP3+ Treg cells within the tumor and significantly increasing the proportion of CD3+CD8+IFN-γ+ Teff cells, thereby achieving dual regulation of the TIME (170). Additionally, the combination of ferroptosis-induced PDT and PD-1 antibodies can activate long-term immune memory and inhibit distant tumor metastasis (144).

Those preclinical studies suggest that combining ferroptosis induction with PD-1/PD-L1 blockade in combination with a multifunctional nanomaterial platform can synergistically reverse the immunosuppressive microenvironment of GBM. However, there is currently no clinical evidence that supports the antitumor effects of ferroptosis-targeted therapies in GBM. The deepening understanding of the immune regulatory mechanisms of ferroptosis and advancements in nanotechnology, combined approach, is expected to provide new therapeutic hope for GBM patients.

## 5 Clinical challenges and future perspectives

### 5.1 Challenges of tumor heterogeneity in the ferroptosis treatment response

GBM exhibits significant molecular heterogeneity, which directly affects the efficacy of ferroptosis-targeted therapies. From a genetic background perspective, TP53 mutation status significantly affects ferroptosis sensitivity by regulating the TP63/GPX4 axis, whereas CDKN2A deletion increases ferroptosis susceptibility by altering the lipid compartment distribution of PUFAs (61, 133).

The spatiotemporal heterogeneity of the TIME further increases treatment complexity. Variations in blood supply across different regions lead to uneven oxygen partial pressure and nutrient distribution, thereby affecting the efficacy of antioxidant defense systems. For example, hypoxic regions upregulate SLC7A11 expression by activating the PI3K/AKT/HIF-1α signaling pathway, enhancing resistance to ferroptosis (117). Metabolic heterogeneity is equally significant, with distinct lipid metabolic profiles, iron ion homeostasis, and antioxidant capacities among different cell subpopulations, resulting in varied responses to interventions such as System Xc^-^ inhibition and GPX4 targeting. This multilayered heterogeneity network necessitates that ferroptosis-targeted therapies adopt precise stratification strategies, integrating technologies such as single-cell sequencing and spatial metabolomics to identify sensitive subpopulations and implement targeted interventions.

### 5.2 Development of ferroptosis biomarkers and precision treatment strategies

The development of reliable ferroptosis biomarkers is a critical step toward achieving personalized treatment. Ideal biomarkers should accurately reflect the sensitivity of tumor cells to ferroptosis and dynamically monitor treatment response during therapy. Current research focuses on several potential biomarkers, including the expression levels of FSP1, FTH1, SLC1A5, NCOA4, and ACSL4; the activity of SLC7A11/GPX4; lipid peroxidation products such as MDA; and iron metabolism-related indicators and ferritinophagy levels. Patient stratification strategies based on molecular features such as CDKN2A deletion, xCT expression, and IDH mutation status have demonstrated predictive value but still require validation through large-scale clinical trials.

In terms of detection technologies, noninvasive imaging techniques and liquid biopsies show great potential. Iron-sensitive probes and lipid peroxidation sensors enable visualization of ferroptosis processes via positron emission tomography (PET) or magnetic resonance imaging (MRI). Liquid biopsies analyze extracellular vesicles (EVs) in blood to detect ferroptosis-associated proteins and nucleic acid biomarkers, offering minimally invasive methods for treatment response assessment. For example, exosomal GPX4, FTH1, and specific miRNA profiles may serve as surrogate indicators of ferroptosis activation. Future research should focus on integrating multiomics biomarkers to construct ferroptosis sensitivity prediction models and validate their clinical utility through prospective clinical trials, ultimately enabling biomarker-guided personalized ferroptosis treatment strategies.

### 5.3 Dynamic microenvironment remodeling and therapy resistance mechanisms

After ferroptosis induction, the TIME undergoes a complex dynamic remodeling process and activates multiple compensatory resistance mechanisms. EVs play a critical role in this process. GBM cells transfer ferroptosis stress to neighboring cells by releasing exosomes rich in ferritin, GPX4, and antioxidant substances while transmitting resistance signals.

Metabolic adaptation is another important resistance mechanism. Under cystine deprivation stress, GBM cells can upregulate GGT1 expression and utilize alternative pathways to maintain GSH homeostasis, thereby preventing the occurrence of ferroptosis (106). The feedback regulation of the TIME further increases complexity. DAMPs released during the initial phase of ferroptosis can activate DCs and T cells, but sustained infiltration of immune cells, particularly M2-type macrophages, may lead to the establishment of an adaptive immunosuppressive environment through the secretion of antioxidant factors and growth factors. To address these dynamic changes, future therapeutic strategies should consider sequential interventions and combination targeting. For example, exosome secretion inhibitors can be administered in a timely manner after ferroptosis inducer treatment or combined with metabolic modulators to block compensatory pathways. Simultaneously, corresponding treatments for dynamic changes in the TME, combined with nanotechnology to achieve controlled drug release, are expected to overcome adaptive resistance.

### 5.4 Safety and selectivity challenges in ferroptosis therapy

Achieving tumor-specific ferroptosis induction while preserving normal tissues, particularly brain function, is a core challenge for clinical translation. Although the BBB limits the ability of most drugs to enter the brain, it also provides a natural barrier to protect normal brain tissue. Astrocytes and oligodendrocytes, which are highly metabolically active cells in the brain, may experience disrupted iron homeostasis, leading to impaired normal neural function, especially given their high sensitivity to oxidative stress. While ferroptosis inducers can increase sensitivity to radiotherapy and chemotherapy, their nonspecific generation of ROS can damage normal neurons and astrocytes. For instance, clinical drugs such as dihydroartemisinin (171), artesunate (172), sorafenib (173), and sulfasalazine (117) show antitumor effects in GBM/glioma by inducing ferroptosis. However, they pose a high risk of dose-dependent neurotoxicity (174, 175). Dihydroartemisinin can significantly restrain neurite outgrowth from differentiating NB2a cells, while this toxic effect could be reversed by L-cysteine, glutathione, and N-acetyl-L-cysteine (176). Hesperetin, a flavone present in fruits and vegetables with neuroprotective effects, can prevent sorafenib-mediated neurotoxicity by relieving brain mitochondrial dysfunction (177). Therefore, those studies provide potential strategies for overcoming drug-induced neurotoxicity by applying neuroprotective agents.

Strategies to improve treatment selectivity include leveraging GBM-specific physiological and molecular characteristics. Compared with other CNS cells, endothelial cells of the BBB and glioma cells show higher levels of TfR1. By utilizing this characteristic, NPs are designed to traverse the BBB through receptor-mediated transcytosis by binding to TfR1, thus achieving specific targeting and killing of glioma tumor cells (178, 179). For example, Zuo developed an H-ferritin (HFn) coated nanoplatform that loads L820 (a conjugate of lonidamine) and IR820 with mitochondrial affinity. This functional material is featured by TfR1-mediated BBB penetration and GBM targeting, and it exhibits significant antitumor effects by mediating profound mitochondrial dysfunction (180). Similarly, Liang et al. developed a self-assembled nanoplatform encapsulating dihydroartemisinin and indocyanine green (ICG, a photosensitizer) onto lactoferrin (LF). By recognizing low-density lipoprotein receptor-related protein-1 (LRP1) on the BBB, this material induces ferroptosis of GBM cells and improves the survival of tumor-bearing mice (181).

Drugs that respond to the TIME to specific stimuli, such as pH-sensitive or matrix metalloproteinase-activated systems, can also increase local drug activation (157, 182). Higher glutathione synthetase (GSS) was an adverse prognostic indicator in patients with glioma, which can prevent radiotherapy-induced ferroptosis in glioma cells. Liu et al. constructed a GSS gene-targeted strategy using the Cas9 protein/sgRNA complex. This complex was loaded into extracellular vesicles (EVs) modified by angiopep-2 (Ang) and a trans-activator of transcription (TAT) peptide. The encapsulating EVs effectively permeated the BBB and penetrated the tumor, finally showing high GSS gene editing efficiency in GBM cells (183). The utilization of endogenous carriers such as exosomes with natural penetration capabilities is another promising strategy (184). Additionally, adjusting dosing regimens is crucial. For example, the use of low-dose, sustained ferroptosis induction rather than high-dose bolus administration may exploit differences in metabolic adaptability between tumor cells and normal cells to improve the therapeutic window.

Future research should focus on developing more precise medicines for regulating ferroptosis, such as conditionally activated prodrug design and bispecific antibody-mediated targeted delivery. Moreover, a deeper understanding of the susceptibility and recovery capacity of different brain cell types to ferroptosis will provide important guidance for optimizing treatment regimens.

### 5.5 Clinical translation of ferroptosis-targeted therapies against GBM

Preclinical studies have shown that ferroptosis inducers (such as erastin and RSL3), sensitize tumor cells to ferroptosis and induce anticancer effects in GBM (185, 186). Despite these encouraging results, the transition of preclinical findings into clinical use has been impeded, possibly due to potential toxicity to healthy tissues (especially the brain and kidney), poor solubility, and metabolic instability (187, 188). Since neurons are enriched with PUFAs, neurons may be more sensitive to ferroptotic stimuli. In mouse stem cell‐derived motor neurons, RSL3 treatment caused significant alterations of synaptogenesis and calcium signaling. However, edaravone, an FDA-approved neuroprotective drug, can prevent RSL3‐induced ferroptosis (189). Similarly, raloxifene is a selective estrogen receptor modulator, and it exerts neuroprotective effects against RSL3-mediated ferroptosis in cultured HT22 neuronal cells (190). LUHMES conditionally immortalized human dopaminergic neurons, which are widely used as a cellular PD model, exhibited enhanced lipid peroxide levels following RSL3 and ML210 treatments. The administration of Fer-1 can prevent neurotoxicity that is induced by ferroptosis inducers (191). At tumoricidal concentrations, RSL3 exhibits cytotoxicity toward normal cells—including neurons, fibroblasts, and nephrocytes—and demonstrates poor selectivity and pharmacokinetic properties because its reactive alkyl chloride group forms a covalent bond with GPX4 (192).

Till now, there is limited clinical evidence of ferroptosis-targeted therapies in cancer treatment. Several ongoing clinical trials are currently conducted. For example, CNSI-Fe is an innovative anti-cancer drug with Fe^2+^ as the active ingredient. Preclinical studies have found that it exhibits anti-tumor functions by regulating the ferroptosis pathway (193). The antitumor effects of CNSI-Fe have been carried out in a first-in-human phase I clinical trial of dose escalation in subjects with advanced solid tumors in China (NCT07433283). Carbon nanoparticle-loaded iron [CNSI-Fe (II)] is considered as a promising antitumor agent due to its unique properties of ferroptosis. A Phase 1 clinical trial of CNSI-Fe(II) has been performed for patients with advanced solid tumors (NCT06048367). TMZ has been found to mediate ferroptosis inhibition and chemoresistance of GBM, and up-regulate dopamine D2 receptor (DRD2) expression. Preclinical studies have suggested that the combination of TMZ and DRD2 antagonists (eg. perphenazine and haloperidol) restrained GBM growth in an orthotopic xenograft model (152, 194). A Phase 2 clinical study is currently conducted to evaluate the effectiveness of haloperidol and TMZ Synergism on adult recurrence GBM (NCT06218524). Consequently, upcoming research should aim to improve drug distribution, minimize systemic toxicity, and optimize the pharmacokinetics of existing ferroptosis inducers.

## 6 Conclusion

The interactions between ferroptosis and the immunosuppressive microenvironment in GBM constitute a complex regulatory network. This review systematically describes how GBM-specific iron metabolism disorders, lipid remodeling, and antioxidant defense imbalances collectively affect tumor susceptibility to ferroptosis and reveal the bidirectional regulatory mechanisms underlying this process and antitumor immune microenvironment remodeling. These findings not only deepen the understanding of the biological behavior of GBM but also provide a theoretical basis for the development of novel therapeutic strategies.

Targeting ferroptosis provides a new therapeutic approach to overcome immune escape in GBM. Ferroptosis inducers enhance DC maturation and CD8^+^ T-cell activation by promoting ICD, releasing DAMPs, and downregulating immune checkpoint molecules, thereby reversing the immunosuppressive microenvironment. When combined with PD-1/PD-L1 blockade, photoimmunotherapy, or metabolic interventions, ferroptosis can further amplify antitumor immune responses. Nanoparticle-based codelivery strategies achieve spatiotemporal synergy between ferroptosis inducers and immunomodulators through precise drug release control, potentially overcoming BBB limitations and reducing systemic toxicity, thereby improving clinical translation feasibility. Additionally, the synergistic mechanism of ferroptosis and ferroptosis offers a novel direction for multimetal ion regulation therapy, providing new hope for recurrent or resistant GBM.

Although ferroptosis-targeted therapy has promising prospects, its clinical translation still faces multiple challenges. Tumor heterogeneity, neurotoxic effects of ferroptosis inducers, BBB penetration efficiency, and treatment specificity are key issues that require resolution. Achieving sufficient tumor selectivity while sparing normal brain tissue remains a critical unresolved challenge for ferroptosis-based therapies in GBM. Future research should focus on developing reliable biomarkers for patient stratification, optimizing the timing and dosage of combination therapies, and enhancing targeting precision and safety through the use of intelligent nanocarriers. Through multidisciplinary collaboration and the integration of innovative technology, personalized ferroptosis-targeted therapeutic strategies may offer new hope for GBM patients.

## Glossary

ACSL4: Acyl-CoA synthetase long chain family member4
ALOXs: Arachidonate-lipoxygenase
BA: Betulinic Acid
BBB: Blood-brain barrier
CA9: Carbonic anhydrase IX
CAFs: Cancer associated fibroblasts
CDKN2A: Cyclin-Dependent Kinase Inhibitor 2A
CoQ10: Coenzyme Q10
CTL: Cytotoxic T Lymphocyte
DAMP: Damage-associated molecular patterns
DC: Dendritic Cell
DHODH: Dihydroorotate Dehydrogenase
DMT1: Divalent Metal Transporter 1
ECH1: Enoy-CoA hydratase 1
EMT: Epithelial-mesenchymal transition
EVs: Extracellular Vesicles
Fabp7: Fatty Acid Binding Protein 7
FADS2: Fatty acid desaturase 2
FAO: Fatty acid oxidation
Fer-1: Ferrostatin-1
IFI16: Interferon-inducible protein 16
LLMs: Lipid-laden macrophages
LPCAT3: Lyso-PL Acyl-transferases 3
MCAD: Medium-chain acyl-CoA dehydrogenase
MDA: Malondialdehyde
MDSC: Myeloid-derived suppressor cells
MGMT: O 6-methylguanine-DNA methyltransferase
MXRA8: Matrix Remodeling Associated 8
NCOA4: Nuclear Receptor Coactivator 4
NK: Natural Killer cells
PCD: Programmed cell death
PD-1: Programmed death 1
PD-L1: Programmed Cell Death Ligand 1
PDT: Photodynamic therapy
PHD: Prolyl Hydroxylase
PLA2G7: Lipoprotein-associated phospholipaseA2
ROS: Reactive Oxygen Species
RSL3: RAS-selective lethal 3
SIRT3: Silent mating type information regulation2 homolog-3
FIN56: Ferroptosis inducer 6
FRG: Ferroptosis-Related Genes
FSP1: Ferroptosis-suppressor-protein 1
FTH1: Ferritin Heavy Chain
FTL: Ferritin Light Chain
GaM: Gallium maltolate
GBM: Glioblastoma
GDEs: GBM cell-derived exosome
GDF15: Growth Differentiation Factor 15
GGT1: Gamma-Glutamyl Transferase 1
GPX4: Glutathione peroxidase 4
GSC: Glioblastoma stem cells
GSH: Glutathione
HFE: Homeostatic iron regulator Gene
HIF: Hypoxia-inducible factor
HO-1: Heme oxygenase 1
I3P: Indole-3-pyruvic acid
ICB: Immune checkpoint blockade
ICD: Immunogenic cell death
IDH: Isocitrate dehydrogenase
IL4I1: Interleukin-4induced 1 protein
STAT1: Signal Transducer and Activator of Transcription 1
System Xc⁻: Cystine/glutamate antiporter system
TAM: Tumor-associated macrophage
TAN: Tumor associated neutrophils
TDP-43: TAR DNA binding protein-43
TfR1: Transferrin receptor protein 1
TME: Tumor immune microenvironment
TMZ: Temozolomide
TRAF3: TNF receptor associated factor 3
Treg: Regulatory T cells
VEGFA: Vascular endothelial growth factor
